# High Conservation of the Human Metapneumovirus Fusion Protein in Contemporary Global Sequences Supports Its Continued Relevance for Therapeutic and Prophylactic Consideration

**DOI:** 10.3390/microorganisms14102255

**Published:** 2026-10-04

**Authors:** Vancheswaran Gopalakrishnan, Emma Schaefer, John Patterson, Tyler Brady, Joseph R. Francica, Anna S. Kushnir, Ondrej Podlaha, Katie Streicher, Taylor S. Cohen, Anastasia A. Aksyuk, Kevin M. Tuffy

**Affiliations:** 1 Bioinformatics, Infectious Disease, BioPharmaceuticals R&D, AstraZeneca, Gaithersburg, MD 20878, USA; vancheswaran.gopalakrishnan@astrazeneca.com (V.G.); john.patterson@astrazeneca.com (J.P.); ondrej.podlaha@astrazeneca.com (O.P.); 2 Translational Medicine, Infectious Disease, BioPharmaceuticals R&D, AstraZeneca, Gaithersburg, MD 20878, USA; emma.schaefer@astrazeneca.com (E.S.); tyler.brady@astrazeneca.com (T.B.); anna.kushnir@astrazeneca.com (A.S.K.); katie.streicher@astrazeneca.com (K.S.); anastasia.aksyuk@astrazeneca.com (A.A.A.); 3 Infectious Disease, BioPharmaceuticals R&D, AstraZeneca, Gaithersburg, MD 20878, USA; joe.francica@astrazeneca.com (J.R.F.); taylor.cohen@astrazeneca.com (T.S.C.)

**Keywords:** human metapneumovirus, molecular surveillance, viral sequencing, fusion protein, gene sequencing, structural modelling

## Abstract

Human metapneumovirus (hMPV) is associated with severe disease in young children, older adults, and medically high-risk individuals. The hMPV F protein is highly immunogenic and a well-established target of neutralizing antibodies. In-depth hMPV surveillance data remain limited and may provide valuable insight into therapeutic development. Here we assembled a contemporary dataset of F gene sequences acquired from hMPV-positive nasal swab specimens collected between 2022 and 2023, together with contemporary F gene sequences from public surveillance databases, collected between 2017 and 2025. All sequences were subtyped and evaluated for residue-level similarity to reference alleles. Of 724 sequences in the combined contemporary dataset, A2 was the most predominant subtype accounting for nearly half of all sequences (50.83%). The mean F gene sequence similarity to reference alleles was 99.90%, 99.95%, and 99.84% for A2, B1, and B2, respectively; the least conserved residues were the stalk residue 518 for A2 (88.52% similarity to reference allele); BinD residue 450 for B1 (96.60% similarity); and site III residue 36 for B2 (77.88% similarity). Structural analysis showed that observed mutations were predominantly surface-exposed, with no evidence of novel N-linked glycosylation sequons, and were not predicted to significantly alter F protein structure. Overall, these findings indicate that the F protein is highly conserved across subtypes, with sequence variation unlikely to result in meaningful structural differences, thereby supporting development of F-targeted therapeutics against hMPV infection.

## 1. Introduction

Human metapneumovirus (hMPV) is a *Pneumoviridae* family viral respiratory pathogen that causes seasonal respiratory tract infections (RTIs). Due to its role in severe disease in young children, older adults, and medically high-risk individuals, hMPV is recognized as a significant global public health burden [1,2,3,4,5]. hMPV has been estimated to be associated with 6.7% of RTIs in children <5 years of age, including ~8000 deaths in 2018 [6,7]. In 2019 alone there were ~473,000 hMPV-associated hospitalizations in adults ≥65 years of age [3]. Additionally, hMPV infections in individuals with immunocompromising conditions more frequently require hospitalization [8,9].

*Pneumoviridae* family viruses have a negative-sense, single-stranded RNA genome encoding nine proteins [10]. Among these, the fusion (F) and attachment surface glycoproteins (G) facilitate the binding and fusion of viral particles to host cells [10]. The G gene is the primary source of genetic variation in the hMPV genome, and this variability underlies the classification of the two major genotypes, A and B, as well as the four subtypes A1, A2, B1, and B2 [10,11,12,13].

Over the last decade, two duplications have been observed in the G gene of hMPV A2 [14,15,16], that have become dominant among the A2 subtype [16]. The increased prevalence of these variants also suggests that they may confer a fitness advantage. hMPV B2 strains containing small 3–12 nucleotide insertions in the same region of G as the A2 duplications have also emerged during this period [17].

While genomic and evolutionary studies have primarily focused on G, the F gene has also been utilized in genotyping and phylogenetic analysis [13,16,18,19]. Unlike the G protein, the F protein is highly immunogenic and is known to be a key target of naturally occurring hMPV neutralizing antibodies [20,21,22,23,24,25]. For these reasons, it is essential to continually monitor the genetic variability of F to ensure that it remains an ideal target for candidate therapeutics.

In this study, we characterized the hMPV F gene in contemporary nasal swab specimens collected in the United States of America (USA) between 2022 and 2023 and integrated these data with a curated global public surveillance dataset of F gene sequences spanning multiple years and countries. Using phylogenetic and comparative genomic analyses, we assessed subtype circulation in contemporary (2017–2025) and historical strains (1982–2016), evaluated geographic and temporal trends in subtype circulation, quantified amino acid conservation, and characterized emerging variations and their structural implications across major hMPV subtypes. Together, these data provide a contemporary framework for understanding hMPV F gene diversity relevant to surveillance and intervention design.

## 2. Materials and Methods

### 2.1. Data Sources

#### 2.1.1. Contemporary Nasal Swab Specimens

F gene sequences were derived from 76 hMPV-positive remnant nasal swab specimens collected at two USA sites, in New York and New Mexico between 2022 and 2023, sourced from Discovery Life Sciences (Huntsville, AL, USA). Viral genetic material from nasal swab specimens was amplified as described in Supplemental Methods. F gene amplicons were sequenced at Azenta Life Sciences (South Plainfield, NJ, USA) using the Illumina (San Diego, CA, USA) 2 × 150 bp platform.

#### 2.1.2. Public Surveillance

The public surveillance dataset comprised F gene sequences collected from 1982 to 2025 sourced from the National Center for Biotechnology Information (NCBI; *n* = 4079, search terms: human metapneumovirus [taxid 162145], has proteins (fusion protein, fusion glycoprotein, fusion, fusion Protein) and the Bacterial and Viral Bioinformatics Resource Center (BV-BRC; *n* = 5065, search terms: F, F protein, F Protein, fusion, fusion protein, fusion Protein, Fusion protein, fusion glycoprotein). Access date: 28 April 2025. We included only F sequences that were at least 500 amino acids in length, yielding 1510 of 4079 sequences from NCBI and 1675 of 5065 sequences from BV-BRC. Of the BV-BRC sequences, 1501 had accessions overlapping with the NCBI dataset and were therefore excluded. For overlapping sequences, metadata from NCBI was given precedence. The resulting surveillance dataset comprised 1684 non-redundant sequences from both databases. After excluding 38 sequences containing ambiguous bases, the final analysis dataset consisted of 1646 sequences (Figure 1). A table of publicly acquired accessions is provided in the Supplementary Materials. Contemporary public sequences were defined as those collected after 2016.

#### 2.1.3. Combined Contemporary Dataset

The combined contemporary dataset comprised public F gene sequences collected from 2017 to 2025 and sequences obtained from hMPV-positive nasal swab specimens.

### 2.2. Bioinformatics

Publicly queried contemporary F gene sequences were de novo aligned with legacy laboratory GenBank reference strains (A1 ref: 00-1 [accession: NC_039199.1], A2 ref: NL/00/17 [accession: FJ168779.1], B1 ref: NL/1/99 [accession: AY525843.1], B2 ref: NL/94/01 [accession: FJ168778.1]) using Augur (v 25.2.0) [26] and phylogenetic trees were constructed using IQ-TREE 2 (v.2.3.5) [27]. The model finder plus algorithm was used to determine the best substitution model for the phylogenetic tree at 1000 bootstraps. Subtyping was based on minimum phylogenetic distance to each reference. To compare the concordance between hMPV subtypes assigned using F gene, G gene, or whole genome sequences, 295 independent contemporary hMPV genomes were obtained from NCBI. F and G genes were extracted using BLASTn (v2.5.0+) and BEDtools (v2.26.0). The F gene, G gene, and whole-genome alignments were processed using the same phylogenetic workflow. Alignments were generated using Augur, and trees were inferred using IQ-TREE 2 with ModelFinder-selected models, 1000 ultrafast bootstrap replicates, and 1000 SH-aLRT replicates. Subtypes were assigned according to the minimum phylogenetic distance to the candidate subtype references.

Selection pressure analysis was performed on the combined contemporary dataset. Two sequences (PP683455, PQ226465) were excluded because their codons were not in frame. The remainder were translated, aligned at the amino-acid level with MAFFT (v7), and back-translated to a codon-aware nucleotide alignment. A maximum-likelihood phylogeny was inferred with IQ-TREE 2, with the best-fit substitution model selected by ModelFinder and support from 1000 ultrafast bootstrap replicates and SH-aLRT tests. Selection was analyzed in HyPhy (v2.5.101): a gene-wide dN/dS ratio (ω) was estimated under the MG94 × REV codon model, and site-specific selection was tested at every codon using the Fixed Effects Likelihood (FEL) method, which contrasts independent synonymous (α) and non-synonymous (β) substitution rates by a likelihood-ratio test (1 d.f.). Sites were considered under selection at *p* ≤ 0.1.

Sequenced amplicons derived from hMPV-positive nasal swab specimens were first trimmed using trimmomatic (v0.39) to remove low-quality regions [28]. Raw reads were then aligned against the reference for each subtype using bwa-mem2 (v 2.3) with subtypes assigned based on the highest average alignment score. Per-position coverage was assessed from the mapping-quality-filtered alignments (mapQ > 30). Across all 76 samples, no uncovered positions were observed, and 100% of reference positions were covered at ≥10,000×. The mean of the sample-level mean depths was 863,955× and the minimum overall depth was 169,225×. Accordingly, no minimum-depth filter was applied during consensus generation. To generate a consensus, an mpileup file was first generated using SAMtools (v 1.6) and converted into a Variant Call Format file using BCFtools (v 1.16) [29,30]. Finally, the consensus was derived from the Binary Alignment Map file using BCFtools-consensus. Only reads that aligned at mapQ score > 30 were retained for generating the consensus. No ambiguous nucleotide codes were detected in the final consensus sequences.

The next-generation sequencing-derived consensus sequences were combined with publicly available contemporary hMPV-F sequences for downstream variant-calling. The most prevalent contemporary sequences (accessions: LC337770, LC337715, MW221988 for A2, B1, and B2, respectively) were used as the references for each subtype to determine circulating variants. After excluding any sequences without a canonical start site, variant calling was done using Remote Analysis of Drug Activity and Resistance (RADAR), an internally developed alignment and variant calling analysis tool, as previously described [31,32]. RADAR is an assembly-/consensus-based variant caller that identifies differences between each input sequence and the relevant reference. Because it operates on assembled or consensus sequences rather than read-level data, it reports consensus-level substitutions and does not detect within-sample minor variants or estimate allele frequencies.

### 2.3. Structural Analysis

The trimeric F protein for each of the contemporary hMPV references was predicted using Boltz-2, version 2.2.0 [33]. To bias towards a pre-fusion conformation, the signal peptide and C-terminal stalk were removed from the reference sequence before prediction. The output model confidence and complex predicted local distance difference test for A2, B1, and B2 were; 0.80:0.82, 0.78:0.81, and 0.76:0.80, respectively. All models were visualized using ChimeraX (v1.10.1) [34]. Root mean square deviation (RMSD) was calculated between a high quality pre-fusion conformation hMPV F protein (Protein Data Bank entry 7UR4) and the predicted model using the matchmaker command in ChimeraX, and was measured over 431 residues of the predicted F protein chain A [35]. The resulting RMSD for A2, B1, and B2 were; 1.766 Å, 1.532 Å, 1.599 Å. Mutations observed in the four most prevalent variant sequences in each subtype were modelled except for those located in the signal peptide, residues 1–18, and beyond residue ca. 465 along the C-terminal coiled-coil stalk. Antigenic sites were labeled by inspecting binding interfaces of hMPV antibodies (Protein Data Bank entry [associated antibody/Fab fragment]: 7UR4 [MPV467], 4DAG [DS7], 8CW9 [ADI61026, MPE8], 7TL0 [SAN32-2]) or comparing to reported sites [20].

### 2.4. Ethical Approval

The study was performed in accordance with the ethical principles originating from the Declaration of Helsinki and that are consistent with the International Council for Harmonisation/Good Clinical Practice, applicable regulatory requirements, applicable legislation on non-interventional studies/observational studies, and the AstraZeneca policy on Bioethics. Remnant hMPV-positive nasal swab specimens were obtained from Discovery Life Sciences (Huntsville, AL, USA) with waived consent under their ethical approval system; the protocol for collection of human biospecimens was approved by WCG IRB (Approval dates: 10 July 2022 and 10 June 2023).

## 3. Results

### 3.1. Characterization of Contemporary hMPV F Sequences

We curated a global contemporary hMPV database comprising F sequences derived from hMPV-positive nasal swab specimens, combined with publicly available sequences (Figure 1). The majority of the 76 hMPV-positive specimens collected at USA sites between 2022 and 2023 were sourced from young individuals, 36.84% of whom were <2 years of age (Table S1). F amplification (RT-PCR amplification *n* = 39, RT-PCR and nested amplification *n* = 37) and subsequent consensus sequences were obtained from all 76 hMPV-positive nasal swabs specimens. A2 was dominant (*n* = 51, 67.11%), followed by B2 (*n* = 20, 26.32%); B1 was less common (*n* = 5, 6.58%). No A1 sequences were identified.

In total, 1646 F gene sequences (having a minimum amino acid length of 500) collected from 26 countries between 1982 and 2025 were included in the public surveillance dataset. Subtypes were assigned based on the F gene. To validate the F gene-based subtyping scheme, 295 test isolates were independently assigned subtypes using F gene, G gene, and whole-genome sequences. For each isolate, the subtype assignments were matched using the accession identifier. In all cases, the assigned subtype corresponded to the reference subtype with the minimum phylogenetic distance. Operationally, all 295 isolates received identical assignments using the three approaches, resulting in 100% pairwise concordance (Figure S1, Table S2).

Countries collecting >100 sequences included the USA (*n* = 584), Australia (*n* = 217), China (*n* = 143), the Netherlands (*n* = 142), Japan (*n* = 140), and Kenya (*n* = 112) (Figure S2A). A2 was dominant in all countries submitting >100 sequences, except Kenya, where B1 was dominant (Figure S2B). Of the 1137 sequences with a collection date, 90.24% (*n* = 1026), were collected post-2010 (Figure 2A). A2 dominated most years; exceptions included 2017 and 2022, when B1 dominated, and 2019, when B2 dominated. A1 was not observed after 2012 (Figure 2B).

Public contemporary sequences were defined as sequences collected after 2016 (*n* = 648), among which the A2 subtype was the most predominant (48.92%, *n* = 317) (Figure 3A). Thirteen countries were represented in the public contemporary dataset, and the majority of sequences (59.10%, *n* = 383) came from the USA (Figure 3B). A2 was again predominant in most countries, except in Australia (*n* = 150 sequences), where all subtypes were equally prevalent, Brazil (*n* = 6), where A2 and B2 were co-dominant, and Indonesia (*n* = 2), which collected one A2 and one B1 sequence (Figure 3C).

Accordingly, the combined contemporary dataset contained 724 F sequences; 50.83% (*n* = 368) were A2 sequences (Figure S3A). There was no predilection for clade based on the source dataset (public surveillance versus nasal swab specimen) (Figure S3B). We measured pairwise patristic distances among sequences in the combined contemporary dataset. Intrasubtype distances were low across all subtypes (median [IQR]: A2, 0.011 [0.006–0.015]; B1, 0.007 [0.005–0.009]; B2, 0.011 [0.008–0.014] substitutions/site). As expected, these were considerably smaller than the distance between the B1 and B2 sublineages and the distance between the A and B subtypes (Figure S4) suggesting high conservation among circulating subtypes. Additionally, the F gene of contemporary hMPV strains was under strong purifying selection, with a gene-wide dN/dS ratio of 0.04, indicating that non-synonymous substitutions accumulate at roughly 4% the rate of synonymous substitutions. Of the 540 codons analyzed, 400 (74%) were under significant purifying selection and two under diversifying selection (Figure S5). The two positively selected codons were only marginally significant and driven by sites with negligible synonymous variation, with one of the residues reflecting a fixed A versus B subtype difference.

### 3.2. Comparison of Contemporary and Historical hMPV Sequences

The distribution of subtypes in the combined contemporary dataset was similar to the distribution observed in historical hMPV sequences that were collected pre-2017 (*n* = 489) (chi-square *p*-value = 0.156; Figure S6). Overall, A and B subtype prevalence was balanced in the historical dataset, comprising 49.48% (*n* = 242) and 50.51% (*n* = 247) of sequences, respectively. Furthermore, A1 was rare (*n* = 12, 2.45%), whereas A2 accounted for nearly half (*n* = 230, 47.03%) of all sequences. In contrast, B1 and B2 subtypes accounted for 24.54% (*n* = 120) and 25.97% (*n* = 127) of sequences, respectively.

### 3.3. Characterizing F Gene Variants

Overall, the F gene was highly conserved from 1982 to 2025, with all three subtypes maintaining high, ≥99% on average, similarity to reference alleles (Figure 4A). To characterize genomic variants, we retained only sequences with a valid canonical start site. This resulted in 1017 sequences for the temporal analysis and 721 sequences for the combined contemporary dataset. We then identified the most prevalent contemporary F sequence for each subtype by aligning public contemporary sequences (A2, *n* = 315; B1, *n* = 142; B2, *n* = 188) against legacy GenBank reference strains. These sequences were subsequently used as references for downstream variant calling, namely LC337770 for A2, LC337715 for B1, and MW221988 for B2, which comprised 59.68%, 80.99%, and 51.06% of all public sequences, respectively.

In the combined contemporary dataset (*n* = 721), the average similarity to the most prevalent contemporary F gene reference sequences across all amino acid residues was 99.90%, 99.95%, and 99.84% for A2, B1, and B2, respectively, suggesting a high degree of conservation (Figure 4B). Among the least conserved residues were the stalk residue 518 for A2 (88.52% similarity to reference); BinD residue 450 for B1 (96.60% similarity); site III residue 36 for B2 (77.88% similarity) (Figure 4C). When considering F sequence variants, the most prevalent variants after the reference sequence were R518K for A2 (5.19%), R450K for B1 (2.72%), and K142R for B2 (7.21%) (Table 1).

### 3.4. Structural Analysis of Observed Variants

We investigated the potential functional impact of mutations identified in our dataset by computationally predicting the structure of the trimeric F protein for each subtype reference sequence and modelling the mutations present in the four most prevalent variant sequences within each subtype. All observed mutations were surface-exposed, barring those not modelled in the C-terminal stalk or signal peptide, and none introduced novel glycosylation sites (Figure 5). Within the four most prevalent variant sequences reported per subtype, the C-terminal stalk of the F protein had four mutations, while no other antigenic site contained more than two prevalent substitutions.

Based on structural modelling, the L36H mutation in B2, located near the interface between site I and site III, induces a cationic shift in a region of site III that is relatively anionic, potentially altering recognition by some site III binders, such as MPE8 (Figure 5C). While R450K in B1 and D296N in B2 observed in antigenic BinD, are currently lacking structural exemplars, most of the modelled mutations are biophysically synonymous lysine to arginine or vice versa, such as K179 found in B2 at antigenic site V, near MPV467 contacts. Three additional mutations were identified at two residues in or near site Ø: K142R and K179R in B2 and R179K in B1, and, based on predictions, these may affect interactions with known antibodies SAN32-2 and ADI-61026. These findings should be confirmed with functional evaluation in future studies.

## 4. Discussion

This study assembled a large hMPV F gene surveillance dataset of 721 contemporary sequences including 76 sequences derived from hMPV-positive nasal swab specimens and 648 publicly available sequences collected after 2016 and spanning multiple countries. Therefore, we provide a contemporary view of hMPV F gene diversity including circulating variants and their structural implications, while specifically demonstrating the marked conservation of the F protein across circulating strains.

The predominance of A2 in both global surveillance and nasal swab specimens derived datasets is consistent with prior observations that this subtype has become increasingly common in recent circulation [17,36,37]. Although some exceptions were observed, including B1 predominance in Kenya and selected years in which B1 or B2 transiently dominated, the overall picture suggests that A2 has remained the leading subtype in much of recent global circulation.

Accurate and ongoing hMPV surveillance is especially important as it has been shown that hMPV variants can emerge and circulate several years before identification [36]. In the combined contemporary dataset, which comprised both public surveillance and nasal swab specimen derived sequences collected from 2017 to 2025, a substantial portion of sequences (63.66%, 79.59%, and 48.56% for A2, B1, and B2, respectively; Table 1) were identical to the identified contemporary reference sequences. These results are supportive of the theory that once successful variants emerge, they are likely to circulate for extended periods.

Data suggesting that neutralizing immune responses primarily target the F protein, rather than the G protein, have informed efforts to develop hMPV therapeutics [20,23,24,25,38,39,40]. We found that contemporary F gene sequences remained similar to reference alleles, consistent with other studies suggesting that the F protein is highly conserved with mutations that are generally localized to only a subset of residues [12,13,41,42]. For instance, sequencing data from the Elias-Warren et al. (2025) study from a community and clinical settings in Seattle, detected all the prominent substitutions that were observed in this study except for H17N in the signal peptide of A2 [41]. Regardless of the differences in data sets, regional acquisition, and analytical approaches, the similar results obtained from these studies suggest sustained constraint on the F protein over historical and contemporary periods. The strong purifying selection acting across the F gene (dN/dS = 0.04), together with the near-absence of positively selected sites, indicates that the hMPV F protein is subject to substantial functional and structural constraint and is not broadly diversifying under immune pressure in circulating strains. This agrees with earlier research by Kim et al. (2016), which found no positively selected sites in the hMPV F gene [43], and extends their observation to a substantially larger and more recent set of circulating strains.

Structural modelling of the most prevalent contemporary variant sequences revealed that observed mutations are predominantly surface-exposed, with no evidence of novel N-linked glycosylation sequons. Therefore, any antigenic differences are more likely mediated by steric rearrangement rather than glycan-based masking of epitopes, which is an established mechanism of escape [44]. A majority of the reported mutations produce biophysically equivalent residues, which are unlikely to alter function or F protein structure, although the effects on binding of individual antibodies cannot be excluded in the absence of experimental validation. The coiled-coil C-terminus of the F protein has low selective pressure and is predicted to tolerate single mutations while maintaining stable trimeric interface in most cases. One particular mutation, L36H observed in B2 at the interface of antigenic site I and site III (the primary targets of the hMPV-neutralizing antibody response [45]), is computationally predicted to introduce a positively charged histidine into a relatively anionic local environment, which may alter charge complementarity at adjacent site III and site II epitopes, potentially modulating antibody recognition. L36H is also predicted to form a new hydrogen-bond network with N240, N280, H332, and D331 alongside a native salt bridge, which may subtly compact the spatial relationship between site II and site III. Although such packing changes may subtly alter local antigenic site geometry and could affect a subset of neutralizing antibodies targeting site II, site V, and site III [38], significant modulation of antibody binding is unlikely as the residues surrounding this site and the protein overall are markedly conserved across contemporary strains. Nevertheless, the effects of these substitutions on antibody binding and neutralization were not assessed experimentally in this study. Taken together, these findings of high sequence similarity and low predicted impact of emerging mutations on antigenic sites suggest that the F protein represents a potential therapeutic target.

While this study is one of the largest analyses of contemporary hMPV surveillance-based sequencing data, inherent differences arising from the use of different sequencing technologies globally, the small number of sequences from some countries and the large proportion of sequences from the US limit firm interpretations. Furthermore, there were inconsistent numbers of sequences across collection years and many sequences in the public dataset did not have a collection date available. Also, the sequences collected may not reflect total global hMPV cases, as the majority were from studies that were not designed to analyze hMPV. As sequencing is generally only done in a clinical setting, these data do not reflect asymptomatic cases. The findings reflect the available deposited sequences rather than true global population-level prevalence of hMPV. Ongoing surveillance programs may help address these limitations by strengthening geographic and temporal representation and improving understanding of hMPV circulation in the broader community. The predictions of the structure of F variant, and their implications for antibody recognition are based on structural modelling and require experimental validation.

## 5. Conclusions

This work establishes a robust baseline for hMPV surveillance and reveals that the F protein is highly conserved with limited diversity observed across contemporary F proteins. Furthermore, no significant alterations to the F protein structure were predicted by the modelling of the most prevalent contemporary substitutions. Although these findings suggest that substantial changes in the overall antigenicity are unlikely, it is possible that individual mutations could affect the binding of certain antibodies, but this was not directly assessed in the present study. Collectively, these data together with the knowledge from other reports detailing the F protein as the primary target of neutralizing immune response, suggest that it maintains high potential as a target for therapeutic strategies.

## Figures and Tables

**Figure 1:**
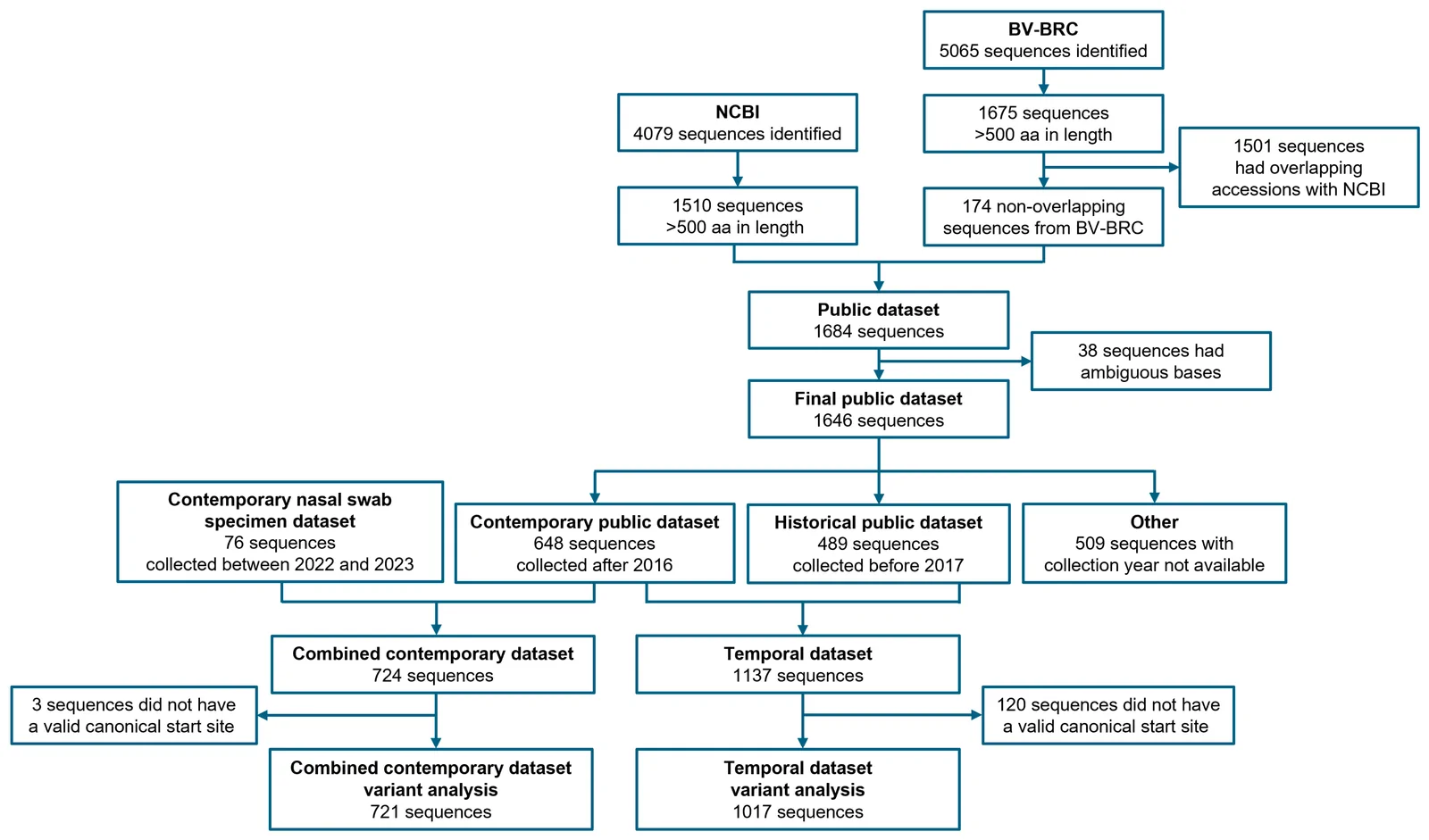
Total number of hMPV sequences in the datasets. aa, amino acid; BV-BRC, Bacterial and Viral Bioinformatics Resource Center; hMPV, human metapneumovirus; NCBI, National Center for Biotechnology Information.

**Figure 2:**
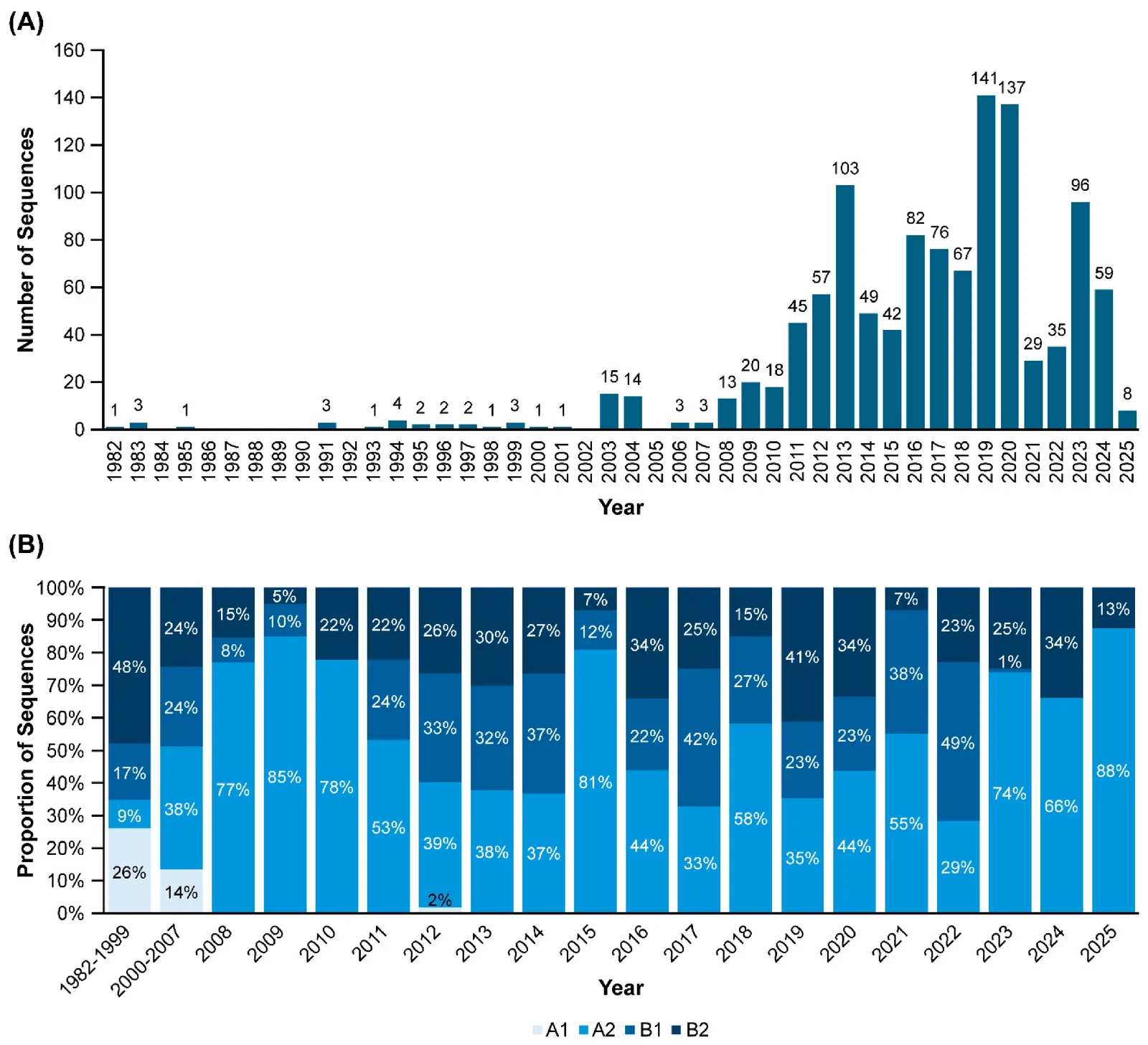
(**A**) Total number of hMPV sequences in the public surveillance dataset between 1982 and 2025 (*n* = 1137 sequences with available collection date) by year of collection, and (**B**) hMPV subtype proportion by year of collection. hMPV, human metapneumovirus.

**Figure 3:**
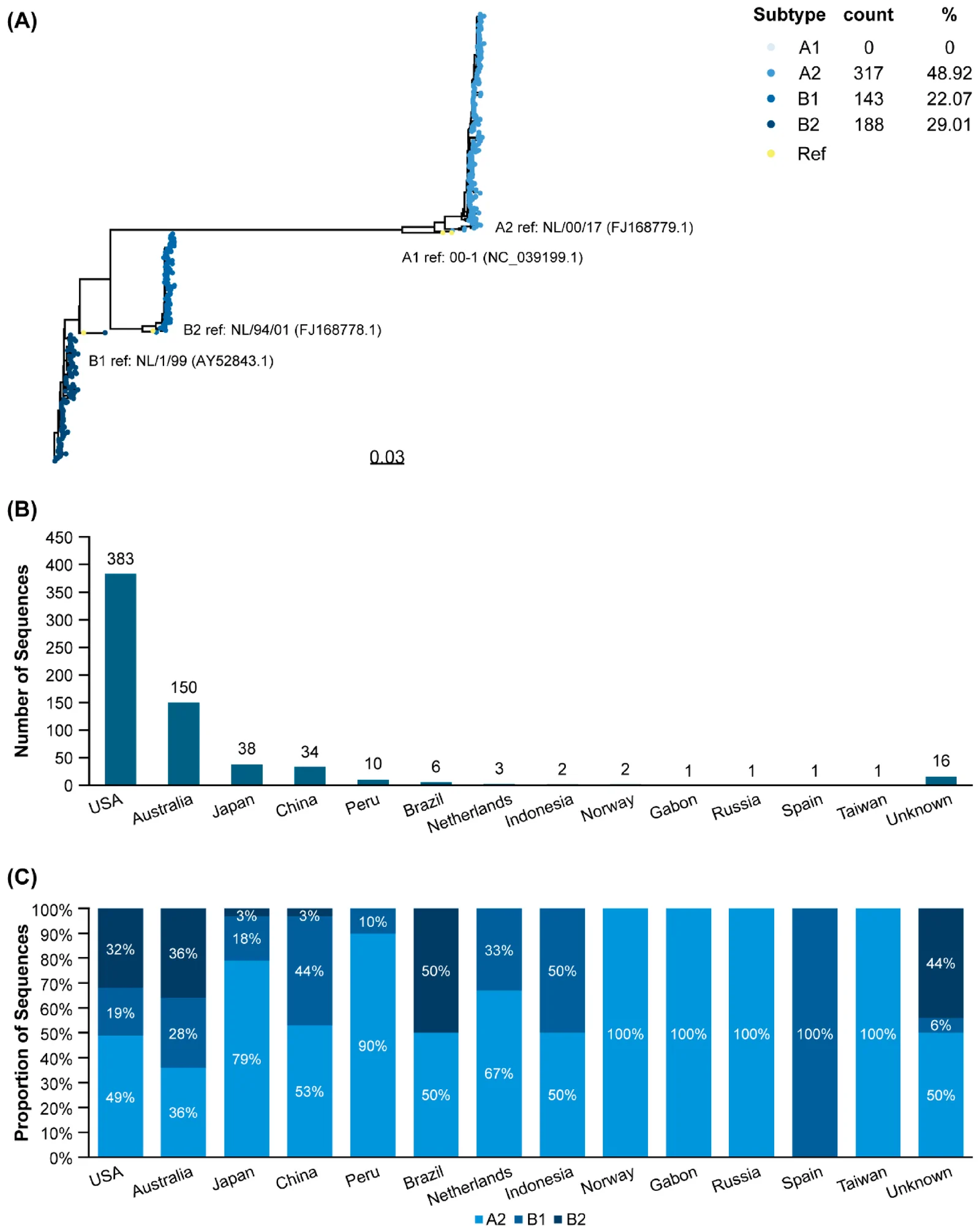
(**A**) Phylogenetic tree of hMPV F gene sequences in the contemporary public dataset between 2017 and 2025 (*n* = 648), (**B**) number of hMPV sequences in the contemporary public dataset by country of origin, and (**C**) hMPV subtype proportion by country of origin in the contemporary public dataset. F, fusion protein; hMPV, human metapneumovirus.

**Figure 4:**
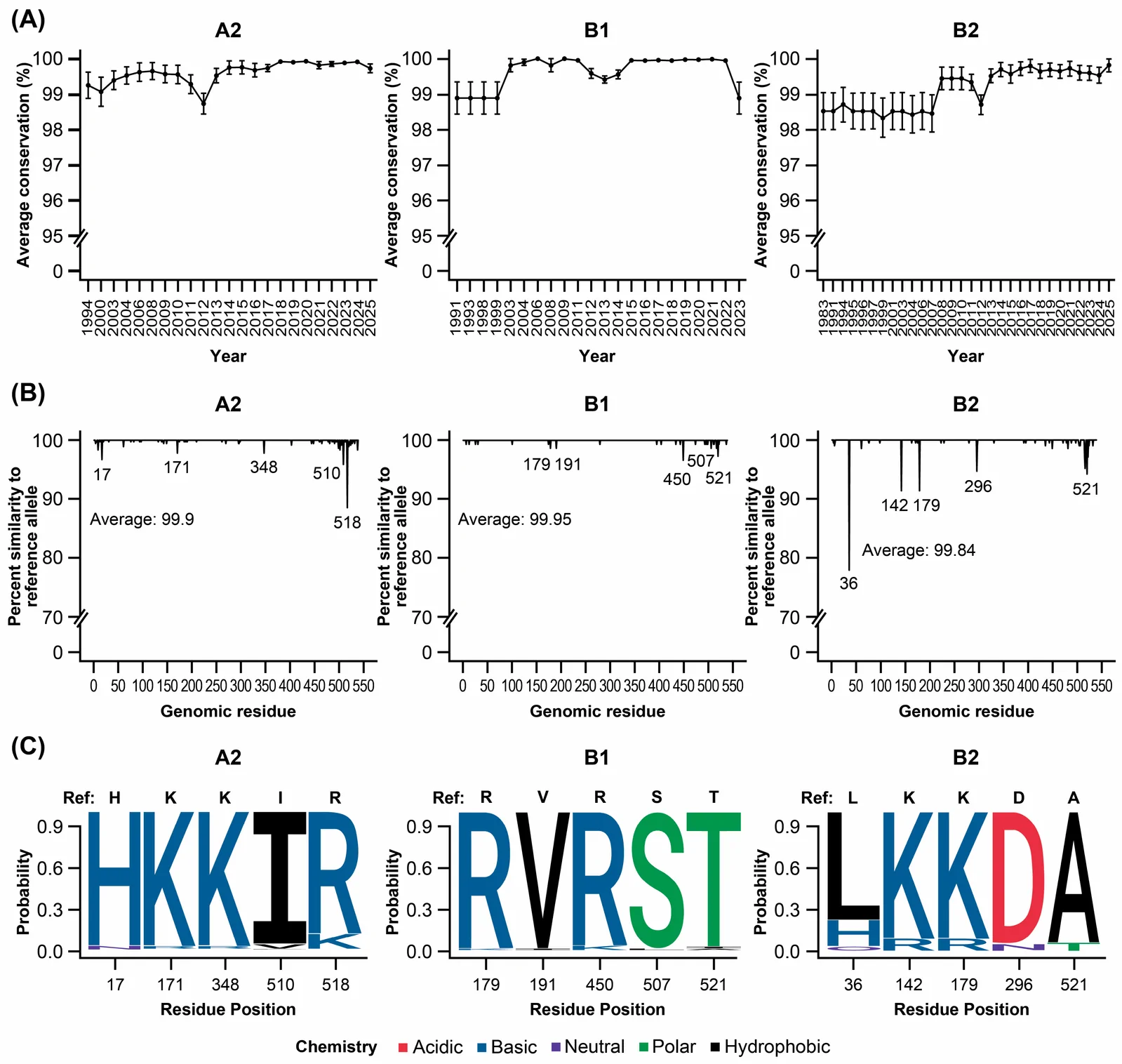
(**A**) hMPV F gene sequence similarity to reference alleles over time in sequences from the public surveillance dataset between 1982 and 2025 (*n* = 1017). (**B**) hMPV F gene sequence similarity to the most prevalent contemporary F gene reference sequences over time in sequences from the combined contemporary dataset (*n* = 721). (**C**) Sequence logo plot showing the distribution of mutations in the top five least conserved residues in all subtypes for sequences within the combined contemporary dataset. The total height of the stack is measured in bits and reflects the information content (i.e., how conserved that position is across sequences at each position). The height of each individual letter within a stack represents the relative frequency of that amino acid at that site. F, fusion protein; hMPV, human metapneumovirus.

**Figure 5:**
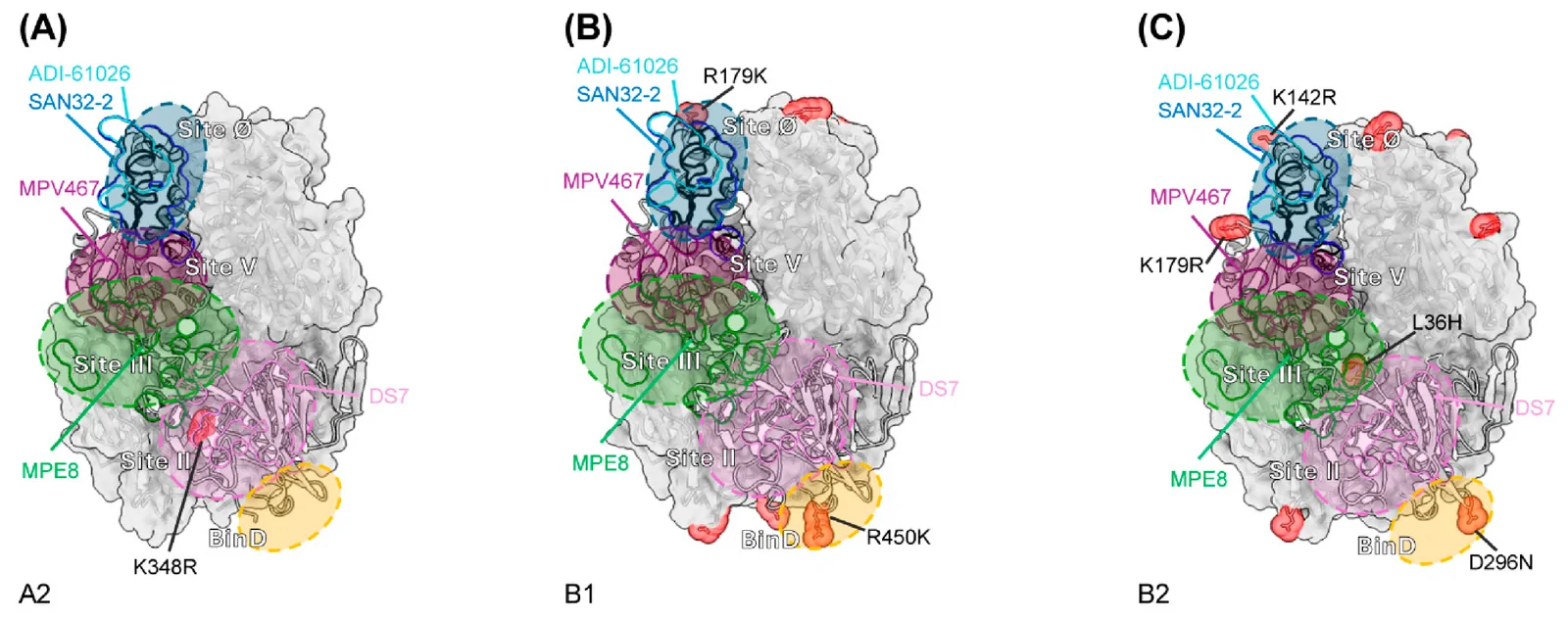
Surface structure of the predicted prefusion conformation of the contemporary F protein references for (**A**) A2, (**B**) B1, and (**C**) B2. Antigenic sites are colored in spheres with known antibody interaction sites outlined. Mutations reported are shown in red and labeled.

**Table 1:** F gene sequence variants within each subtype from the combined contemporary dataset (*n* = 721).

A2			B1			B2		
Variant	Count	%	Variant	Count	%	Variant	Count	%
Reference	233	63.66	Reference	117	79.59	Reference	101	48.56
R518K	19	5.19	R450K	4	2.72	K142R	15	7.21
H17N	8	2.19	R179K	2	1.36	L36H	14	6.73
K348R	8	2.19	S507L	2	1.36	L36H:K179R	10	4.81
I510V:R518K	6	1.64	T521A	2	1.36	D296N	8	3.85
K171R	6	1.64	T521I	2	1.36	T517A:A521T	8	3.85
S503F	4	1.09	V191I:R518K	2	1.36	L36Q	5	2.4
F9L	3	0.82	A523T	1	0.68	L36H:K179R:516insT	4	1.92
T521A	3	0.82	E26K	1	0.68	A523T	3	1.44
A523V	2	0.55	H435Y	1	0.68	G297E:N482S:T517A: A521T	2	0.96
A61S:K143T:S149T:M270T:V449I:R518K	2	0.55	H538Y	1	0.68	G533S	2	0.96
A61T	2	0.55	I13V	1	0.68	L36H:R518K	2	0.96
F511L	2	0.55	I31V	1	0.68	M505I	2	0.96
F9L:R518K	2	0.55	I512V	1	0.68	M6T	2	0.96
I506V	2	0.55	I7V	1	0.68	P525L	2	0.96
I510F	2	0.55	K405R	1	0.68	R450K:A523T	2	0.96
K296R:I510V:R518K	2	0.55	K519R	1	0.68	A415T	1	0.48
N533S	2	0.55	S101P:D280G:R396W: R450K:E475D:I495V	1	0.68	D296N:516insT	1	0.48
P520S	2	0.55	S175G:I513T	1	0.68	D296N:V436I	1	0.48
P525L	2	0.55	S444N	1	0.68	I7V	1	0.48
S482N	2	0.55	V494A	1	0.68	K142R:N223T:R229K: N280D:T392I:Q396R: N444S:R450K:K479R: A521T	1	0.48
S539N	2	0.55	V498A	1	0.68	K142R:V436I	1	0.48
V500I	2	0.55	V500A	1	0.68	K171R	1	0.48
D185N	1	0.27				K4R:L36H	1	0.48
D461E:Q462E:G487E	1	0.27				L36H:A521T	1	0.48
F9L:R518K:S539N	1	0.27				L36H:I498V	1	0.48
G297D	1	0.27				L36H:I506M	1	0.48
G490S	1	0.27				L36H:K179R:A472T: 516insT	1	0.48
G522E	1	0.27				L36H:K179R:D331N	1	0.48
H17N:I495T	1	0.27				L36H:P98H:K179R	1	0.48
H17N:R479K:S503F	1	0.27				L36H:S507L	1	0.48
H17N:S504G	1	0.27				L36H:T61I:K179R:T504A	1	0.48
H17Y	1	0.27				L36H:T61N	1	0.48
I13V:Q94R:K142T:K188L	1	0.27				L36Q:A523P	1	0.48
I31V	1	0.27				N482S	1	0.48
I492T:V500I	1	0.27				P520L	1	0.48
I492V	1	0.27				R163K:D296N	1	0.48
I496V:R518K	1	0.27				R518K	1	0.48
I510V:R518K:P520T	1	0.27				S175N	1	0.48
I510V:R518K:S539N	1	0.27				S507L	1	0.48
I514L	1	0.27				S76N:T489I	1	0.48
K171R:S445N	1	0.27				T61A:K142R	1	0.48
K171R:V500I	1	0.27						
K20R	1	0.27						
K4R:T521A	1	0.27						
K4T:I506V	1	0.27						
K82R	1	0.27						
N210H	1	0.27						
N404S:I510V:R518K	1	0.27						
N404S:I510V:R518K:A523T	1	0.27						
N404S:R518K	1	0.27						
N466S	1	0.27						
N97D:I510V:R518K	1	0.27						
P525L:S539N	1	0.27						
R175S	1	0.27						
R518K:S539N	1	0.27						
S132G	1	0.27						
S445G:A499T	1	0.27						
S503F:R518K	1	0.27						
S528N	1	0.27						
S76N	1	0.27						
T517A	1	0.27						
T517K	1	0.27						
T531I	1	0.27						
V191I	1	0.27						
V262I	1	0.27						
V500I:R518K	1	0.27						
V508M	1	0.27						
V5E:G297E	1	0.27						
V6A:S10A:N139S	1	0.27						
Y299H:N466S:S528N	1	0.27						

The most prevalent contemporary sequences were used as the reference for each subtype (accessions: LC337770, LC337715, MW221988 for A2, B1, and B2, respectively). ins, insertion.

## Data Availability

Data underlying the findings described in this paper may be obtained in accordance with AstraZeneca’s data sharing policy described at https://astrazenecagrouptrials.pharmacm.com/ST/Submission/Disclosure, accessed on 23 September 2026. Data for studies directly listed on Vivli can be requested through Vivli at www.vivli.org. Data for studies not listed on Vivli could be requested through Vivli at https://vivli.org/members/enquiries-about-studies-not-listed-on-the-vivli-platform/, accessed on 23 September 2026. The AstraZeneca Vivli member page is also available, outlining further details: https://vivli.org/ourmember/astrazeneca/, accessed on 23 September 2026. The 4079 unique sequences identified in the NCBI and BV-BRC searches are publicly available. The 76 sequences derived from hMPV-positive remnant nasal swab specimens are available from NCBI GenBank upon publication of this manuscript (NCBI GenBank accession numbers: QB094070–QB094145).

## Supplementary Materials

The following supporting information can be downloaded at: https://www.mdpi.com/article/10.3390/microorganisms14102255/s1, Supplementary Table—Accessions; Supplemental Methods; Table S1. Characteristics of patients from whom nasal swab specimens were collected for the contemporary nasal swab specimen dataset (*n* = 76); Table S2. Subtype concordance based on F gene, G gene, and whole genome. The assigned subtype corresponded to the reference subtype with the minimum distance; Figure S1. Pairwise agreement matrices comparing hMPV F gene-based subtype assignments with G gene- and whole-genome-based assignments; Figure S2. (A) Total number of hMPV sequences in the public surveillance dataset between 1982 and 2025 (*n* = 1646) by country of origin, and (B) hMPV subtype proportion by country of origin; Figure S3. Phylogenetic trees of (A) all hMPV F gene sequences in the combined contemporary data set (*n* = 724), and (B) based on source of sequences (contemporary public surveillance dataset versus contemporary nasal swab specimen dataset); Figure S4. Pairwise phylogenetic distances among contemporary hMPV F sequences; Figure S5. Per-codon selection across the hMPV F gene estimated by FEL (*n* = 722; IQ-TREE 2 phylogeny); Figure S6. hMPV subtype distribution in historical (pre-2017, *n* = 489^a^) and contemporary (*n* = 724) datasets.

## Abbreviations

The following abbreviations are used in this manuscript:

BV-BRC	Bacterial and Viral Bioinformatics Resource Center
F	fusion protein
G	glycoprotein
hMPV	human metapneumovirus
NCBI	National Center for Biotechnology Information
RMSD	root mean square deviation
RTI	respiratory tract infection
