# Supplementary material for: High Conservation of the Human Metapneumovirus Fusion Protein in Contemporary Global Sequences Supports Its Continued Relevance for Therapeutic and Prophylactic Consideration
Source: Microorganisms. 2026 Oct 4;14(10):2255. doi: 10.3390/microorganisms14102255 (PMC13707668; doi:10.3390/microorganisms14102255)
Supplement: Supplementary file 1 [file microorganisms-14-02255-s001.zip › hMPV_sequencing_Supplement_21Sep26.pdf]

Supplementary Materials

High Conservation of the Human Metapneumovirus Fusion Protein in Contemporary Global Sequences Supports Its Continued Relevance for Therapeutic and Prophylactic Consideration

Contents

Supplemental Methods 2

Table S1. Characteristics of patients from whom nasal swab specimens were collected for the contemporary nasal swab specimen dataset (*N* = 76). 4

Table S2. Subtype concordance based on F gene, G gene, and whole genome. The assigned subtype corresponded to the reference subtype with the minimum distance........ 5

Figure S1. Pairwise agreement matrices comparing hMPV F gene-based subtype assignments with G gene- and whole-genome-based assignments........ 11

Figure S2. (A) Total number of hMPV sequences in the public surveillance dataset between 1982 and 2025 (*N* = 1646) by country of origin, and (B) hMPV subtype proportion by country of origin. 12

Figure S3. Phylogenetic trees of (A) all hMPV F gene sequences in the combined contemporary data set (*N* = 724), and (B) based on source of sequences (contemporary public surveillance dataset versus contemporary nasal swab specimen dataset). 13

Figure S4. Pairwise phylogenetic distances among contemporary hMPV F sequences........ 14

Figure S5. Per-codon selection across the hMPV F gene estimated by FEL (*N* = 722; IQ-TREE 2 phylogeny). 15

Figure S6. hMPV subtype distribution in historical (pre-2017, *N* = 489[a]) and contemporary (*N* = 724) datasets........ 16

Supplemental Methods

Amplification of Contemporary Nasal Swab Specimen Viral Genetic Material

Viral genetic material from collected nasal swab specimens were amplified and sequenced using the 2x150bp protocol on the Illumina platform. Viral ribonucleic acid (vRNA) was isolated from nasal swab samples using QIAamp Viral RNA mini kit (Cat. No. 52906, Qiagen, Valencia, CA, USA) according to manufacturer's protocol. A one-step reverse transcriptase polymerase chain reaction (RT-PCR) was conducted using Superscript IV One-Step RT-PCR System (Cat. No. 12594025, ThermoFisher Scientific, Waltham, MA, USA) reagents and hMPV specific (F2-2 forward [5'-TGAACAATCCMAAAGGYATATTCAAG-3'] and R3-2 reverse [5'-CTRCCTGCKCCTGATATTATTG-3'] primer sequences. Reactions contained 25 µL of 2X Platinum SuperFi Master Mix, 2.5 µL of each primer, 0.5 µL of Superscript IV RT, 15 µL of template RNA, and 4.5 µL of H_2O. Thermal cycler conditions were 50°C for 20 minutes, 98°C for 3 minutes, 5X 98°C for 10 seconds, 55°C for 10 seconds, and 72°C for 70 seconds followed by 40X 98°C for 10 seconds, 59°C for 10 seconds, 72°C for 70 seconds, and 72°C for 5 minutes. Samples were run on a gel to ensure the correct band size (~2.2 kb). 45 µL of each reaction was gel extracted using QIAquick Gel Extraction Kit (Cat. No. 28704, Qiagen) and quantitated using the TapeStation DNA ScreenTape System (Agilent, Santa Clara, CA, USA).

For samples with insufficient complementary DNA (cDNA) for next-generation sequencing (less than 20 ng/µL or 500 ng total), the remaining DNA material was further processed via a nested PCR. Nested PCR was conducted utilizing Q5 Hot Start High-Fidelity System (M0494S, NEB, Ipswich, MA, USA) reagents and nested primer sequences (F5-2 forward [5'-AGRATMTGCAAGASYTGGAGCC-3'] and R1-2 reverse [5'-CGYACTTCATATTTGCATGGAGC-3']). Reactions contained 25 µL of Q5 HotStart Master Mix, 2.5 µL of each primer, 2 µL of DNA, and 18 µL of H_2O. Thermal cycler conditions were

98°C for 3 minutes, 30X 98°C for 10 seconds, 59°C for 30 seconds, 72°C for 55 seconds, and 72°C for 2 minutes. Samples were run on a gel to ensure the correct band size, gel extracted and quantitated as above.

Table S1. Characteristics of patients from whom nasal swab specimens were collected for the contemporary nasal swab specimen dataset (*N* = 76).

**Patient characteristics**	***n* (%)**
Age category, years	
<2	28 (36.84)
2–18	16 (21.05)
18–60	13 (17.11)
>60	19 (25.00)
Gender	
Female	39 (51.32)
Male	37 (48.68)
Race	
Native American	1 (1.32)
Asian	1 (1.32)
Black	14 (18.42)
Other	1 (1.32)
Unknown	30 (39.47)
White	29 (38.16)

Table S2. Subtype concordance based on F gene, G gene, and whole genome. The assigned subtype corresponded to the reference subtype with the minimum distance.

Accession	Sub-type	A1 distance	A2 distance	B1 distance	B2 distance	Sub-type	A1 distance	A2 distance	B1 distance	B2 distance	Sub-type	A1 distance	A2 distance	B1 distance	B2 distance	Concordant
LC789936.1	A2	0.108911732	0.052679132	0.4765541	0.466606276	A2	0.388965519	0.152147616	2.04484574	2.018229973	A2	0.098776111	0.047067254	0.390870271	0.375968653	TRUE
LC817384.1	B1	0.483313688	0.485233052	0.043283412	0.106912962	B1	1.892056122	2.107558842	0.101645889	0.320940637	B1	0.378662901	0.387045383	0.035427211	0.083380895	TRUE
LC817385.1	B2	0.474989831	0.476909195	0.10853693	0.033630229	B2	1.859573866	2.075076585	0.315074148	0.118576218	B2	0.371964342	0.380346824	0.091583954	0.028953339	TRUE
LC817386.1	B2	0.473019876	0.47493924	0.106566975	0.031660274	B2	1.866583876	2.082086595	0.322084158	0.125586228	B2	0.368185923	0.376568404	0.087805535	0.02517492	TRUE
LC823194.1	A2	0.115997671	0.059765071	0.483640039	0.473692215	A2	0.398743746	0.161925843	2.054623967	2.0280082	A2	0.102505288	0.05079643	0.394599448	0.37969783	TRUE
LC823195.1	A2	0.11638699	0.060154391	0.484029359	0.474081534	A2	0.403282979	0.166465076	2.059163201	2.032547434	A2	0.103754385	0.052045528	0.395848545	0.380946927	TRUE
LC889306.1	A2	0.11550656	0.05927396	0.483148928	0.473201104	A2	0.405482172	0.168664269	2.061362394	2.034746627	A2	0.106888336	0.055179478	0.398982496	0.384080878	TRUE
LC889307.1	A2	0.116352552	0.060119953	0.483994921	0.474047096	A2	0.407736133	0.17091823	2.063616355	2.037000588	A2	0.104381731	0.052672873	0.396475891	0.381574273	TRUE
LC889308.1	A2	0.116723104	0.060490504	0.484365472	0.474417648	A2	0.414174942	0.177357039	2.070055164	2.043439397	A2	0.105000739	0.053291882	0.397094899	0.382193281	TRUE
LC889309.1	A2	0.115306807	0.059074207	0.482949175	0.473001351	A2	0.406287867	0.169469964	2.062168089	2.035552322	A2	0.105001641	0.053292784	0.397095801	0.382194183	TRUE
LC889310.1	B2	0.475050144	0.476969508	0.108597243	0.033690542	B2	1.839233518	2.054736237	0.2947338	0.09823587	B2	0.367027768	0.37541025	0.08664738	0.024016765	TRUE
LC889311.1	B2	0.47551222	0.477431584	0.109059319	0.034152618	B2	1.845259602	2.060762322	0.300759884	0.104261954	B2	0.366399426	0.374781908	0.086019039	0.023388423	TRUE
MH150888.1	A2	0.097358842	0.002224687	0.46500121	0.455053386	A2	0.377178122	0.008361785	2.033058344	2.006442577	A2	0.080338194	0.000620889	0.372432354	0.357530735	TRUE
MH150889.1	A2	0.097433177	0.002299023	0.465075546	0.455127721	A2	0.377178122	0.008361785	2.033058344	2.006442577	A2	0.080338194	0.000620889	0.372432354	0.357530735	TRUE
MK087726.1	A2	0.108457341	0.052224741	0.476099709	0.466151885	A2	0.381995358	0.145177455	2.03787558	2.011259813	A2	0.096907241	0.045198384	0.389001401	0.374099783	TRUE
MK167039.2	A2	0.106327232	0.050094633	0.473969601	0.464021776	A2	0.378200165	0.141382262	2.034080387	2.00746462	A2	0.095642735	0.043933878	0.387736895	0.372835277	TRUE
MK167040.2	A2	0.1084782	0.052245601	0.476120569	0.466172744	A2	0.386578351	0.149760447	2.042458572	2.015842805	A2	0.096280353	0.044571495	0.388374513	0.373472894	TRUE
MK820375.1	B1	0.477802454	0.479721818	0.037772178	0.101401729	B1	1.861007064	2.076509783	0.07059683	0.289891579	B1	0.374911942	0.383294424	0.031676252	0.079629936	TRUE
MN306019.1	B1	0.479095611	0.481014975	0.039065335	0.102694886	B1	1.874426973	2.089929692	0.08401674	0.303311488	B1	0.378120836	0.386503318	0.034885146	0.08283883	TRUE
MN306024.2	A2	0.110154081	0.053921481	0.477796449	0.467848624	A2	0.382846256	0.146028352	2.038726477	2.01211071	A2	0.100666461	0.048957603	0.392760621	0.377859003	TRUE
MN306028.2	A2	0.10962185	0.05338925	0.477264218	0.467316394	A2	0.382846256	0.146028352	2.038726477	2.01211071	A2	0.100666461	0.048957603	0.392760621	0.377859003	TRUE
MN745084.1	A2	0.108452063	0.052219463	0.476094431	0.466146607	A2	0.382000232	0.145182329	2.037880454	2.011264686	A2	0.099409867	0.047701009	0.391504027	0.376602408	TRUE
MN745085.1	A2	0.107941738	0.051709138	0.475584106	0.465636281	A2	0.392720218	0.155902315	2.04860044	2.021984672	A2	0.098142085	0.046433227	0.390236245	0.375334627	TRUE
MN745086.1	A2	0.108101098	0.051868498	0.475743466	0.465795642	A2	0.387448664	0.150630761	2.043328886	2.016713119	A2	0.096907899	0.045199042	0.389002059	0.374100441	TRUE
MN745087.1	A2	0.109742779	0.053510179	0.477385147	0.467437323	A2	0.395919509	0.159101606	2.051799731	2.025183964	A2	0.098770076	0.047061218	0.390864236	0.375962617	TRUE
MZ221202.1	B1	0.478590266	0.480509631	0.038559991	0.102189541	B1	1.866077952	2.081580671	0.075667718	0.294962467	B1	0.375532593	0.383915074	0.032296902	0.080250587	TRUE
MZ851793.1	A2	0.109574286	0.053341686	0.477216654	0.467268829	A2	0.390379999	0.153562096	2.046260221	2.019644454	A2	0.096901267	0.04519241	0.388995427	0.374093809	TRUE
MZ851794.1	A2	0.109877325	0.053644726	0.477519694	0.467571869	A2	0.39454049	0.157722587	2.050420712	2.023804945	A2	0.096287701	0.044578844	0.388381861	0.373480243	TRUE
MZ851795.1	A2	0.10898031	0.052747711	0.476622679	0.466674854	A2	0.441820249	0.205002346	2.097700471	2.071084704	A2	0.098158027	0.046449169	0.390252187	0.375350569	TRUE
MZ851796.1	A2	0.109232163	0.052999563	0.476874531	0.466926707	A2	0.385205934	0.148388031	2.041086156	2.014470389	A2	0.100020318	0.04831146	0.392114478	0.377212859	TRUE
MZ851797.1	A2	0.11181408	0.055581481	0.479456449	0.469508624	A2	0.387308902	0.150490999	2.043189124	2.016573357	A2	0.098768248	0.047059391	0.390862408	0.37596079	TRUE
MZ851992.1	A2	0.108774515	0.052541915	0.476416883	0.466469059	A2	0.391663527	0.154845624	2.047543748	2.020927981	A2	0.096288889	0.044580032	0.388383049	0.373481431	TRUE
MZ851993.1	A2	0.10887727	0.05264467	0.476519638	0.466571814	A2	0.378902649	0.142084746	2.034782871	2.008167104	A2	0.100635216	0.048926358	0.392729376	0.377827757	TRUE
OK644703.1	B1	0.479903755	0.481823119	0.039873479	0.10350303	B1	1.867868171	2.08337089	0.077457937	0.296752686	B1	0.379294415	0.387676897	0.036058725	0.084012409	TRUE
OL794368.1	B2	0.469480487	0.471399852	0.103027586	0.028120886	B2	1.832255995	2.047758714	0.287756277	0.091258347	B2	0.365353242	0.373735724	0.084972854	0.022342239	TRUE
OL794375.1	A2	0.111694188	0.055461588	0.479336556	0.469388732	A2	0.41053138	0.173713477	2.066411602	2.039795835	A2	0.101763117	0.050054259	0.393857277	0.378955659	TRUE
OL794378.1	A2	0.109374429	0.05314183	0.477016798	0.467068973	A2	0.3679792	0.131161297	2.023859422	1.997243654	A2	0.102546854	0.050837996	0.394641014	0.379739395	TRUE
OL794383.1	B1	0.476803258	0.478722622	0.036772982	0.100402532	B1	1.869325149	2.084827868	0.078914915	0.298209664	B1	0.37531534	0.383697822	0.03207965	0.080033334	TRUE
OL794387.1	B2	0.469363739	0.471283103	0.102910838	0.028004137	B2	1.845548277	2.061050996	0.301048559	0.104550629	B2	0.365651339	0.374033821	0.085270951	0.022640336	TRUE
OL794404.1	A2	0.109008189	0.052775589	0.476650557	0.466702733	A2	0.382242281	0.145424378	2.038122503	2.011506736	A2	0.099405706	0.047696849	0.391499866	0.376598248	TRUE
OL794405.1	B2	0.470605173	0.472524537	0.104152272	0.029245571	B2	1.847124753	2.062627472	0.302625035	0.106127105	B2	0.36565385	0.374036332	0.085273462	0.022642847	TRUE
OL794407.1	A2	0.110115695	0.053883095	0.477758064	0.467810239	A2	0.397737706	0.160919803	2.053617928	2.02700216	A2	0.102720542	0.051011684	0.394814702	0.379913084	TRUE
OL794418.1	A2	0.10978135	0.053548751	0.477423719	0.467475894	A2	0.378647989	0.141830086	2.034528211	2.007912444	A2	0.102549262	0.050840405	0.394643422	0.379741804	TRUE
OL794419.1	A2	0.111526388	0.055293788	0.479168756	0.469220932	A2	0.389304995	0.152487092	2.045185217	2.01856945	A2	0.099397947	0.04768909	0.391492107	0.376590489	TRUE
OL794431.1	A2	0.109521061	0.053288461	0.477163429	0.467215605	A2	0.386088792	0.149270889	2.041969014	2.015353247	A2	0.095698821	0.043989964	0.387792981	0.372891363	TRUE
OL794432.1	B2	0.468914598	0.470833962	0.102461697	0.027554996	B2	1.833087746	2.048590465	0.288588028	0.092090097	B2	0.367759373	0.376141855	0.087378985	0.02474837	TRUE
OL794443.1	A2	0.108643647	0.052411047	0.476286015	0.46633819	A2	0.387328041	0.150510138	2.043208263	2.016592496	A2	0.100646565	0.048937708	0.392740725	0.377839107	TRUE

Accession	Sub-type	A1 distance	A2 distance	B1 distance	B2 distance	Sub-type	A1 distance	A2 distance	B1 distance	B2 distance	Sub-type	A1 distance	A2 distance	B1 distance	B2 distance	Concordant
OL794455.1	A2	0.110839336	0.054606736	0.478481704	0.46853388	A2	0.39424679	0.157428887	2.050127011	2.023511244	A2	0.10328656	0.051577703	0.39538072	0.380479102	TRUE
OL794466.1	B2	0.467808727	0.469728092	0.101355827	0.026449126	B2	1.817640068	2.033142787	0.27314035	0.07664242	B2	0.368440534	0.376823016	0.088060147	0.025429531	TRUE
OL794475.1	B2	0.469916522	0.471835886	0.103463621	0.02855692	B2	1.843678365	2.059181084	0.299178646	0.102680716	B2	0.365019583	0.373402064	0.084639195	0.02200858	TRUE
OL794476.1	B2	0.468390754	0.470310119	0.101937853	0.027031153	B2	1.811520628	2.027023347	0.26702091	0.07052298	B2	0.365210755	0.373593236	0.084830367	0.022199752	TRUE
OL794483.1	B2	0.468927245	0.47084661	0.102474345	0.027567644	B2	1.842485481	2.0579882	0.297985763	0.101487833	B2	0.364392943	0.372775425	0.084012556	0.02138194	TRUE
OM262393.1	A2	0.110069642	0.053837042	0.47771201	0.467764186	A2	0.387448664	0.150630761	2.043328886	2.016713119	A2	0.10001848	0.048309622	0.39211264	0.377211022	TRUE
OM262394.1	A2	0.109003895	0.052771295	0.476646263	0.466698439	A2	0.387448664	0.150630761	2.043328886	2.016713119	A2	0.097532492	0.045823634	0.389626652	0.374725034	TRUE
OM262395.1	A2	0.109435814	0.053203215	0.477078183	0.467130358	A2	0.381995358	0.145177455	2.03787558	2.011259813	A2	0.097531145	0.045822287	0.389625305	0.374723687	TRUE
OM262396.1	A2	0.110241373	0.054008773	0.477883741	0.467935917	A2	0.3934714	0.156653497	2.049351622	2.022735855	A2	0.098149746	0.046440888	0.390243906	0.375342288	TRUE
OM262397.1	A2	0.110451246	0.054218646	0.478093614	0.46814579	A2	0.395919509	0.159101606	2.051799731	2.025183964	A2	0.100026126	0.048317268	0.392120286	0.377218667	TRUE
OM262398.1	A2	0.108573715	0.052341115	0.476216083	0.466268258	A2	0.395021029	0.158203125	2.05090125	2.024285483	A2	0.097527161	0.045818303	0.389621321	0.374719702	TRUE
OM262399.1	A2	0.108573715	0.052341115	0.476216083	0.466268258	A2	0.395021029	0.158203125	2.05090125	2.024285483	A2	0.097527161	0.045818303	0.389621321	0.374719702	TRUE
OM262400.1	A2	0.108585245	0.052352646	0.476227614	0.466279789	A2	0.390133219	0.153315316	2.046013441	2.019397674	A2	0.097524784	0.045815926	0.389618944	0.374717325	TRUE
OM262401.1	A2	0.108572726	0.052340126	0.476215094	0.46626727	A2	0.395021029	0.158203125	2.05090125	2.024285483	A2	0.097527161	0.045818303	0.389621321	0.374719702	TRUE
OM262402.1	A2	0.108732908	0.052500308	0.476375276	0.466427452	A2	0.391713222	0.154895319	2.047593444	2.020977677	A2	0.098153582	0.046444724	0.390247742	0.375346124	TRUE
OM262403.1	A2	0.109172862	0.052940262	0.47681523	0.466867406	A2	0.38356972	0.146751817	2.039449942	2.012834175	A2	0.098151853	0.046442995	0.390246013	0.375344395	TRUE
OM262404.1	A2	0.109317121	0.053084521	0.476959489	0.467011664	A2	0.385168672	0.148350769	2.041048893	2.014433126	A2	0.096287701	0.044578844	0.388381861	0.373480243	TRUE
OM262405.1	A2	0.108959199	0.052726599	0.476601568	0.466653743	A2	0.390682387	0.153864484	2.046562609	2.019946842	A2	0.097866205	0.046157347	0.389960365	0.375058746	TRUE
OM262406.1	A2	0.108321557	0.052088958	0.475963926	0.466016101	A2	0.383651104	0.146833201	2.039531326	2.012915559	A2	0.098776429	0.047067571	0.390870589	0.37596897	TRUE
OM262407.1	A2	0.109839707	0.053607107	0.477482075	0.467534251	A2	0.383569577	0.146751674	2.039449799	2.012834031	A2	0.097525445	0.045816588	0.389619605	0.374717987	TRUE
OM262408.1	A2	0.109648055	0.053415455	0.477290423	0.467342599	A2	0.38515003	0.148332127	2.041030252	2.014414485	A2	0.098152218	0.04644336	0.390246378	0.375344759	TRUE
OM262409.1	A2	0.109801608	0.053569008	0.477443976	0.467496152	A2	0.391398347	0.154580444	2.047278569	2.020662802	A2	0.098763267	0.047054409	0.390857427	0.375955808	TRUE
OM262410.1	A2	0.109996904	0.053764304	0.477639272	0.467691448	A2	0.394799595	0.157981692	2.050679816	2.024064049	A2	0.097525853	0.045816995	0.389620013	0.374718395	TRUE
OM262411.1	A2	0.108013402	0.051780803	0.475655771	0.465707946	A2	0.391572898	0.154754995	2.04745312	2.020837353	A2	0.097793428	0.04608457	0.389887588	0.37498597	TRUE
OM262412.1	A2	0.110432494	0.054199894	0.478074862	0.468127038	A2	0.395516015	0.158698112	2.051396237	2.02478047	A2	0.099409558	0.047700701	0.391503718	0.3766021	TRUE
OM262413.1	A2	0.109415048	0.053182448	0.477057416	0.467109592	A2	0.383573178	0.146755275	2.0394534	2.012837633	A2	0.096902457	0.045193599	0.388996617	0.374094998	TRUE
OM262414.1	A2	0.109723276	0.053490676	0.477365644	0.46741782	A2	0.385161532	0.148343629	2.041041754	2.014425987	A2	0.096287701	0.044578844	0.388381861	0.373480243	TRUE
OM262415.1	A2	0.109523858	0.053291258	0.477166226	0.467218402	A2	0.391579856	0.154761953	2.047460078	2.02084431	A2	0.100015167	0.048306309	0.392109327	0.377207708	TRUE
OM262416.1	A2	0.110144981	0.053912381	0.477787349	0.467839524	A2	0.385133093	0.14831519	2.041013315	2.014397548	A2	0.09815377	0.046444912	0.39024793	0.375346311	TRUE
OM262417.1	A2	0.109263895	0.053031295	0.476906263	0.466958439	A2	0.377300852	0.140482949	2.033181074	2.006565307	A2	0.099131698	0.04742284	0.391225858	0.37632424	TRUE
OM262418.1	B2	0.471206173	0.473125538	0.104753273	0.029846572	B2	1.829966853	2.045469572	0.285467135	0.088969204	B2	0.365157983	0.373540465	0.084777595	0.02214698	TRUE
ON168639.1	B1	0.476920428	0.478839793	0.036890153	0.100519703	B1	1.862729815	2.078232534	0.072319582	0.29161433	B1	0.376790315	0.385172797	0.033554625	0.081508309	TRUE
ON729327.1	B1	0.476210363	0.478129727	0.036180087	0.099809637	B1	1.869349011	2.08485173	0.078938777	0.298233526	B1	0.373664419	0.382046901	0.030428729	0.078382413	TRUE
OP345076.1	A2	0.110035115	0.053802515	0.477677483	0.467729659	A2	0.448207562	0.211389659	2.104087784	2.077472017	A2	0.098151705	0.046442847	0.390245865	0.375344246	TRUE
OP345077.1	A2	0.109082253	0.052849653	0.476724621	0.466776797	A2	0.387448664	0.150630761	2.043328886	2.016713119	A2	0.100659236	0.048950378	0.392753396	0.377851777	TRUE
OP345078.1	A2	0.109893256	0.053660657	0.477535625	0.4675878	A2	0.395916977	0.159099074	2.051797199	2.025181432	A2	0.098771897	0.047063039	0.390866057	0.375964438	TRUE
OP345079.1	A2	0.109521285	0.053288685	0.477163653	0.467215829	A2	0.395919509	0.159101606	2.051799731	2.025183964	A2	0.098770076	0.047061218	0.390864236	0.375962617	TRUE
OP345080.1	A2	0.109701026	0.053468427	0.477343395	0.46739557	A2	0.395919509	0.159101606	2.051799731	2.025183964	A2	0.098770076	0.047061218	0.390864236	0.375962617	TRUE
OP345081.1	A2	0.110298494	0.054065894	0.477940862	0.467993038	A2	0.384080977	0.147263074	2.039961199	2.013345432	A2	0.098150019	0.046441161	0.390244179	0.375342561	TRUE
OP345082.1	A2	0.110070612	0.053838013	0.477712981	0.467765156	A2	0.384134885	0.147316982	2.040015106	2.013399339	A2	0.100019334	0.048310477	0.392113494	0.377211876	TRUE
OP345083.1	A2	0.111364273	0.055131673	0.479006641	0.469058817	A2	0.387308902	0.150490999	2.043189124	2.016573357	A2	0.096906568	0.04519771	0.389000728	0.37409911	TRUE
OP345084.1	A2	0.109660761	0.053428161	0.477303129	0.467355305	A2	0.384882716	0.148064813	2.040762938	2.014147171	A2	0.098151333	0.046442475	0.390245493	0.375343875	TRUE
OP358284.1	A2	0.108615697	0.052383097	0.476258065	0.466310241	A2	0.392763932	0.155946029	2.048644154	2.022028387	A2	0.097527161	0.045818303	0.389621321	0.374719702	TRUE
OP358285.1	A2	0.11071501	0.054482411	0.478357379	0.468409554	A2	0.416698762	0.179880859	2.072578983	2.045963216	A2	0.097527161	0.045818303	0.389621321	0.374719702	TRUE
OP358286.1	A2	0.107757011	0.051524412	0.47539938	0.465451555	A2	0.38037844	0.143560536	2.036258661	2.009642894	A2	0.100340547	0.048631689	0.392434707	0.377533088	TRUE
OP358287.1	A2	0.109354514	0.053121914	0.476996882	0.467049058	A2	0.386170304	0.149352401	2.042050526	2.015434759	A2	0.09815302	0.046444162	0.39024718	0.375345561	TRUE
OP358288.1	A2	0.111515674	0.055283074	0.479158042	0.469210218	A2	0.387308902	0.150490999	2.043189124	2.016573357	A2	0.096906568	0.04519771	0.389000728	0.37409911	TRUE
PP315925.1	A2	0.108879432	0.052646832	0.4765218	0.466573976	A2	0.387584662	0.150766759	2.043464884	2.016849117	A2	0.096936501	0.045227644	0.389030661	0.374129043	TRUE
PP315926.1	B1	0.477384822	0.479304186	0.037354546	0.100984097	B1	1.859923021	2.07542574	0.069512787	0.288807535	B1	0.372890326	0.381272807	0.029654635	0.07760832	TRUE
PP716093.1	A2	0.115255342	0.059022743	0.482897711	0.472949886	A2	0.407666349	0.170848446	2.063546571	2.036930804	A2	0.103760139	0.052051281	0.395854299	0.38095268	TRUE
PP716094.1	A2	0.115255342	0.059022743	0.482897711	0.472949886	A2	0.407666349	0.170848446	2.063546571	2.036930804	A2	0.103760139	0.052051281	0.395854299	0.38095268	TRUE
PP716095.1	A2	0.114745481	0.058512881	0.482387849	0.472440025	A2	0.394753614	0.15793571	2.050633835	2.024018068	A2	0.105020756	0.053311898	0.397114916	0.382213298	TRUE

Accession	Sub-type	A1 distance	A2 distance	B1 distance	B2 distance	Sub-type	A1 distance	A2 distance	B1 distance	B2 distance	Sub-type	A1 distance	A2 distance	B1 distance	B2 distance	Concordant
PP716096.1	A2	0.115327274	0.059094674	0.482969643	0.473021818	A2	0.397015734	0.160197831	2.052895956	2.026280189	A2	0.10500579	0.053296932	0.39709995	0.382198331	TRUE
PP947553.1	A2	0.110093832	0.053861232	0.477736201	0.467788376	A2	0.385821613	0.149003709	2.041701834	2.015086067	A2	0.099401122	0.047692264	0.391495282	0.376593663	TRUE
PP947554.1	B2	0.469809991	0.471729355	0.10335709	0.028450389	B2	1.812306457	2.027809176	0.267806739	0.071308809	B2	0.363176627	0.371559109	0.08279624	0.020165624	TRUE
PP947555.1	B1	0.478536642	0.480456006	0.038506366	0.102135917	B1	1.876080169	2.091582889	0.085669936	0.304964684	B1	0.376159643	0.384542125	0.032923953	0.080877637	TRUE
PP947556.1	A2	0.110026613	0.053794013	0.477668981	0.467721157	A2	0.389172334	0.152354431	2.045052555	2.018436788	A2	0.099399107	0.047690249	0.391493267	0.376591649	TRUE
PP947557.1	A2	0.11039973	0.05416713	0.478042098	0.468094274	A2	0.381653806	0.144835903	2.037534028	2.010918261	A2	0.100645647	0.04893679	0.392739807	0.377838189	TRUE
PP947558.1	B2	0.471729992	0.473649356	0.105277091	0.03037039	B2	1.85482989	2.070332609	0.310330172	0.113832242	B2	0.366279967	0.374662448	0.085899579	0.023268963	TRUE
PP947559.1	A2	0.11054524	0.054312641	0.478187609	0.468239784	A2	0.394917087	0.158099184	2.050797309	2.024181542	A2	0.100639226	0.048930368	0.392733386	0.377831767	TRUE
PP947560.1	B2	0.469307316	0.47122668	0.102854415	0.027947714	B2	1.823692633	2.039195352	0.279192915	0.082694985	B2	0.36339762	0.371780101	0.083017232	0.020386616	TRUE
PP947561.1	B2	0.471425662	0.473345027	0.104972762	0.030066061	B2	1.850308003	2.065810722	0.305808284	0.109310354	B2	0.367560006	0.375942488	0.087179618	0.024549003	TRUE
PP947562.1	A2	0.110017206	0.053784606	0.477659574	0.46771175	A2	0.388543957	0.151726054	2.044424178	2.017808411	A2	0.100643705	0.048934847	0.392737865	0.377836246	TRUE
PP947563.1	A2	0.110101366	0.053868767	0.477743735	0.46779591	A2	0.389177844	0.152359941	2.045058066	2.018442298	A2	0.100019407	0.04831055	0.392113567	0.377211949	TRUE
PP947564.1	B2	0.47054831	0.472467674	0.104095409	0.029188708	B2	1.833223752	2.048726472	0.288724034	0.092226104	B2	0.365156023	0.373538505	0.084775635	0.02214502	TRUE
PP947565.1	B2	0.471170425	0.47308979	0.104717525	0.029810824	B2	1.850304583	2.065807302	0.305804865	0.109306935	B2	0.36690948	0.375291962	0.086529092	0.023898477	TRUE
PP947566.1	B2	0.472528369	0.474447733	0.106075468	0.031168767	B2	1.851776774	2.067279493	0.307277055	0.110779125	B2	0.368802657	0.377185138	0.088422269	0.025791654	TRUE
PP947567.1	B1	0.480786922	0.482706286	0.040756646	0.104386196	B1	1.864154398	2.079657117	0.073744164	0.293038913	B1	0.37740965	0.385792132	0.03417396	0.082127644	TRUE
PP947568.1	B1	0.480807646	0.48272701	0.04077737	0.104406921	B1	1.868156068	2.083658787	0.077745834	0.297040582	B1	0.376786609	0.385169091	0.033550919	0.081504603	TRUE
PP947569.1	B1	0.480112637	0.482032001	0.040082361	0.103711911	B1	1.857863553	2.073366272	0.067453319	0.286748067	B1	0.376163117	0.384545599	0.032927427	0.080881111	TRUE
PP947570.1	B2	0.470390483	0.472309847	0.103937582	0.029030881	B2	1.836223827	2.051726546	0.291724109	0.095226179	B2	0.364532831	0.372915312	0.084152443	0.021521827	TRUE
PP947571.1	B1	0.478790203	0.480709567	0.038759927	0.102389477	B1	1.859350659	2.074853378	0.068940426	0.288235174	B1	0.375538718	0.383921199	0.032303027	0.080256712	TRUE
PP947572.1	B1	0.480787911	0.482707275	0.040757635	0.104387185	B1	1.864154398	2.079657117	0.073744164	0.293038913	B1	0.377408147	0.385790629	0.034172457	0.082126141	TRUE
PP947573.1	B1	0.477910949	0.479830313	0.037880673	0.101510224	B1	1.864438603	2.079941322	0.074028369	0.293323118	B1	0.374918526	0.383301008	0.031682836	0.07963652	TRUE
PP947574.1	A2	0.110542088	0.054309489	0.478184457	0.468236632	A2	0.394913142	0.158095239	2.050793364	2.024177597	A2	0.10125944	0.049550583	0.3933536	0.378451982	TRUE
PP947575.1	A2	0.110157329	0.053924729	0.477799697	0.467851873	A2	0.39157556	0.154757657	2.047455782	2.020840015	A2	0.100638075	0.048929217	0.392732235	0.377830617	TRUE
PP947576.1	B2	0.470933634	0.472852998	0.104480733	0.029574032	B2	1.848759685	2.064262404	0.304259966	0.107762036	B2	0.366282173	0.374664655	0.085901785	0.02327117	TRUE
PP947577.1	B2	0.471330027	0.473249391	0.104877126	0.029970425	B2	1.848758685	2.064261404	0.304258966	0.107761036	B2	0.365653538	0.374036019	0.08527315	0.022642535	TRUE
PP947578.1	B2	0.471091463	0.473010827	0.104638562	0.029731861	B2	1.85030403	2.065806749	0.305804312	0.109306382	B2	0.366281117	0.374663598	0.085900729	0.023270114	TRUE
PP947579.1	B2	0.471331067	0.473250431	0.104878166	0.029971465	B2	1.850306415	2.065809134	0.305806697	0.109308766	B2	0.366276147	0.374658628	0.085895759	0.023265144	TRUE
PP947580.1	B2	0.471019223	0.472938587	0.104566322	0.029659621	B2	1.861697189	2.077199908	0.317197471	0.120699541	B2	0.366284647	0.374667129	0.085904259	0.023273644	TRUE
PP947581.1	B1	0.480861554	0.482780918	0.040831278	0.104460828	B1	1.864154398	2.079657117	0.073744164	0.293038913	B1	0.37740965	0.385792132	0.03417396	0.082127644	TRUE
PP947582.1	B2	0.470298678	0.472218043	0.103845778	0.028939077	B2	1.850308674	2.065811393	0.305808956	0.109311026	B2	0.36690553	0.375288012	0.086525142	0.023894527	TRUE
PP947583.1	B1	0.479714368	0.481633733	0.039684092	0.103313643	B1	1.859540822	2.075043541	0.069130588	0.288425337	B1	0.376786056	0.385168537	0.033550365	0.08150405	TRUE
PP947584.1	B1	0.480479809	0.482399173	0.040449533	0.104079084	B1	1.869993627	2.085496346	0.079583393	0.298878141	B1	0.376168631	0.384551113	0.032932941	0.080886625	TRUE
PP947585.1	B1	0.479279581	0.481198945	0.039249305	0.102878856	B1	1.867710117	2.083212836	0.077299883	0.296594632	B1	0.379319404	0.387701886	0.036083714	0.084037398	TRUE
PP947586.1	B1	0.480282849	0.482202214	0.040252573	0.103882124	B1	1.862435304	2.077938023	0.07202507	0.291319819	B1	0.379961238	0.38834372	0.036725548	0.084679232	TRUE
PP947587.1	B2	0.470445513	0.472364877	0.103992612	0.029085911	B2	1.848645926	2.064148645	0.304146208	0.107648278	B2	0.366908903	0.375291385	0.086528516	0.0238979	TRUE
PP947588.1	B2	0.471330269	0.473249634	0.104877369	0.029970668	B2	1.847095081	2.0625978	0.302595363	0.106097433	B2	0.367537829	0.37592031	0.087157441	0.024526825	TRUE
PP947589.1	B1	0.480816387	0.482735751	0.040786111	0.104415661	B1	1.873415741	2.08891846	0.083005507	0.302300256	B1	0.378676226	0.387058708	0.035440536	0.08339422	TRUE
PP947590.1	A2	0.110475222	0.054242622	0.47811759	0.468169766	A2	0.391250185	0.154432282	2.047130407	2.02051464	A2	0.10002839	0.048319533	0.39212255	0.377220932	TRUE
PP947591.1	B1	0.478840431	0.480759795	0.038810155	0.102439705	B1	1.864442224	2.079944943	0.07403199	0.293326739	B1	0.37742259	0.385805072	0.0341869	0.082140584	TRUE
PP947592.1	B2	0.470161049	0.472080413	0.103708148	0.028801447	B2	1.835912843	2.051415562	0.291413125	0.094915195	B2	0.363278721	0.371661203	0.082898333	0.020267718	TRUE
PP947593.1	B1	0.478432871	0.480352235	0.038402595	0.102032145	B1	1.86444501	2.079947729	0.074034777	0.293329525	B1	0.376793795	0.385176277	0.033558105	0.081511789	TRUE
PP947594.1	B1	0.477788271	0.479707635	0.037757995	0.101387546	B1	1.869794467	2.085297186	0.079384233	0.298678981	B1	0.374908087	0.383290569	0.031672397	0.079626081	TRUE
PP947595.1	A2	0.110255027	0.054022427	0.477897395	0.467949571	A2	0.387120816	0.150302913	2.043001037	2.01638527	A2	0.10002839	0.048319533	0.39212255	0.377220932	TRUE
PP947596.1	B2	0.471012674	0.472932038	0.104559773	0.029653072	B2	1.848759685	2.064262404	0.304259966	0.107762036	B2	0.365654545	0.374037027	0.085274157	0.022643542	TRUE
PP947597.1	A2	0.109932124	0.053699525	0.477574493	0.467626668	A2	0.384387036	0.147569133	2.040267258	2.013651491	A2	0.100645647	0.04893679	0.392739807	0.377838189	TRUE
PP947598.1	A2	0.107822519	0.051589919	0.475464888	0.465517063	A2	0.389863793	0.15304589	2.045744015	2.019128248	A2	0.097502973	0.045794115	0.389597133	0.374695514	TRUE
PP947599.1	A2	0.111375267	0.055142668	0.479017636	0.469069811	A2	0.385111173	0.14829327	2.040991395	2.014375628	A2	0.103164919	0.051456062	0.395259079	0.380357461	TRUE
PP947600.1	A2	0.110282699	0.0540501	0.477925068	0.467977243	A2	0.394973991	0.158156088	2.050854213	2.024238446	A2	0.098767982	0.047059124	0.390862142	0.375960524	TRUE
PP947601.1	B2	0.471408065	0.473327429	0.104955164	0.030048463	B2	1.848759685	2.064262404	0.304259966	0.107762036	B2	0.366910676	0.375293158	0.086530289	0.023899673	TRUE
PP947602.1	A2	0.109951551	0.053718951	0.477593919	0.467646095	A2	0.387118776	0.150300873	2.042998998	2.016383231	A2	0.099401122	0.047692264	0.391495282	0.376593663	TRUE
PP947603.1	B2	0.471248862	0.473168226	0.104795961	0.02988926	B2	1.851852689	2.067355408	0.307352971	0.110855041	B2	0.366281117	0.374663598	0.085900729	0.023270114	TRUE

Accession	Sub-type	A1 distance	A2 distance	B1 distance	B2 distance	Sub-type	A1 distance	A2 distance	B1 distance	B2 distance	Sub-type	A1 distance	A2 distance	B1 distance	B2 distance	Concordant
PP947604.1	B2	0.471328319	0.473247683	0.104875418	0.029968717	B2	1.848625706	2.064128425	0.304125988	0.107628058	B2	0.367537829	0.37592031	0.087157441	0.024526825	TRUE
PP947605.1	B1	0.479500532	0.481419896	0.039470256	0.103099806	B1	1.884266495	2.099769214	0.093856261	0.313151009	B1	0.377424522	0.385807004	0.034188832	0.082142516	TRUE
PP947606.1	B1	0.478951011	0.480870376	0.038920735	0.102550286	B1	1.86103607	2.076538789	0.070625836	0.289920584	B1	0.376792093	0.385174575	0.033556403	0.081510087	TRUE
PP947607.1	A2	0.110588287	0.054355687	0.478230655	0.468282831	A2	0.38305976	0.146241857	2.038939982	2.012324215	A2	0.100648293	0.048939436	0.392742453	0.377840835	TRUE
PP947608.1	B2	0.471004978	0.472924342	0.104552077	0.029645376	B2	1.851772318	2.067275037	0.307272599	0.110774669	B2	0.369432612	0.377815094	0.089052224	0.026421609	TRUE
PP947609.1	B1	0.479188165	0.481107529	0.039157889	0.10278744	B1	1.882361559	2.097864278	0.091951325	0.311246073	B1	0.376806076	0.385188558	0.033570386	0.08152407	TRUE
PP947610.1	A2	0.110339856	0.054107256	0.477982224	0.4680344	A2	0.381649614	0.144831711	2.037529836	2.010914069	A2	0.100645647	0.04893679	0.392739807	0.377838189	TRUE
PP947611.1	B1	0.479345563	0.481264927	0.039315287	0.102944837	B1	1.882585591	2.09808831	0.092175357	0.311470105	B1	0.376806076	0.385188558	0.033570386	0.08152407	TRUE
PP947612.1	A2	0.11030685	0.054074251	0.477949219	0.468001394	A2	0.381082239	0.144264336	2.036962461	2.010346694	A2	0.101894313	0.050185456	0.393988473	0.379086855	TRUE
PP947613.1	A2	0.110911244	0.054678644	0.478553613	0.468605788	A2	0.387270896	0.150452993	2.043151118	2.016535351	A2	0.100021178	0.04831232	0.392115338	0.37721372	TRUE
PP947614.1	B2	0.470470614	0.472389979	0.104017714	0.029111013	B2	1.833223752	2.048726472	0.288724034	0.092226104	B2	0.365156023	0.373538505	0.084775635	0.02214502	TRUE
PP947615.1	B1	0.479423933	0.481343297	0.039393657	0.103023208	B1	1.884266495	2.099769214	0.093856261	0.313151009	B1	0.376806076	0.385188558	0.033570386	0.08152407	TRUE
PP947616.1	A2	0.107435751	0.051203151	0.475078119	0.465130294	A2	0.381756185	0.144938282	2.037636406	2.011020639	A2	0.097502926	0.045794068	0.389597086	0.374695467	TRUE
PP947617.1	B2	0.471005748	0.472925112	0.104552847	0.029646146	B2	1.844018904	2.059521623	0.299519185	0.103021255	B2	0.365645367	0.374027848	0.085264979	0.022634363	TRUE
PP947618.1	A2	0.111069687	0.054837087	0.478712055	0.468764231	A2	0.383047518	0.146229615	2.03892774	2.012311973	A2	0.10377926	0.052070402	0.39587342	0.380971801	TRUE
PP947619.1	B2	0.471244542	0.473163907	0.104791642	0.029884941	B2	1.844023648	2.059526368	0.29952393	0.103026	B2	0.365646506	0.374028988	0.085266119	0.022635503	TRUE
PP947620.1	A2	0.107924418	0.051691818	0.475566786	0.465618961	A2	0.386327067	0.149509164	2.042207289	2.015591522	A2	0.096884815	0.045175957	0.388978975	0.374077356	TRUE
PP947621.1	B1	0.478405737	0.480325101	0.038375461	0.102005011	B1	1.859351897	2.074854616	0.068941663	0.288236412	B1	0.375538718	0.383921199	0.032303027	0.080256712	TRUE
PP947622.1	A2	0.108967775	0.052735175	0.476610143	0.466662319	A2	0.384390415	0.147572512	2.040270637	2.01365487	A2	0.100018163	0.048309305	0.392112323	0.377210705	TRUE
PP947623.1	B2	0.469389433	0.471308798	0.102936533	0.028029832	B2	1.8236612	2.039163919	0.279161482	0.082663552	B2	0.36339762	0.371780101	0.083017232	0.020386616	TRUE
PP947624.1	B1	0.478714414	0.480633778	0.038684138	0.102313688	B1	1.859350659	2.074853378	0.068940426	0.288235174	B1	0.375538718	0.383921199	0.032303027	0.080256712	TRUE
PP947625.1	B1	0.479962145	0.48188151	0.03993187	0.10356142	B1	1.885949369	2.101452088	0.095539136	0.314833884	B1	0.376806076	0.385188558	0.033570386	0.08152407	TRUE
PP947626.1	B2	0.471973814	0.473893178	0.105520913	0.030614212	B2	1.84710245	2.062605169	0.302602732	0.106104802	B2	0.367541861	0.375924343	0.087161473	0.024530858	TRUE
PP947627.1	B1	0.480787911	0.482707275	0.040757635	0.104387185	B1	1.864154398	2.079657117	0.073744164	0.293038913	B1	0.37740965	0.385792132	0.03417396	0.082127644	TRUE
PP947628.1	A2	0.110616346	0.054383747	0.478258715	0.46831089	A2	0.38098274	0.144164837	2.036862962	2.010247195	A2	0.101262795	0.049553937	0.393356955	0.378455337	TRUE
PP947629.1	B2	0.471408546	0.47332791	0.104955645	0.030048944	B2	1.848759685	2.064262404	0.304259966	0.107762036	B2	0.367532017	0.375914499	0.08715163	0.024521014	TRUE
PP947630.1	B2	0.470855625	0.472774989	0.104402724	0.029496023	B2	1.848759685	2.064262404	0.304259966	0.107762036	B2	0.365653538	0.374036019	0.08527315	0.022642535	TRUE
PP947631.1	A2	0.109894362	0.053661762	0.477536731	0.467588906	A2	0.394490263	0.15767236	2.050370485	2.023754718	A2	0.100640273	0.048931416	0.392734433	0.377832815	TRUE
PP947632.1	A2	0.112458611	0.056226011	0.480100979	0.470153155	A2	0.39147034	0.154652437	2.047350562	2.020734795	A2	0.101920246	0.050211388	0.394014406	0.379112787	TRUE
PP947633.1	A2	0.111620278	0.055387678	0.479262646	0.469314822	A2	0.393551276	0.156733373	2.049431498	2.022815731	A2	0.100656523	0.048947665	0.392750683	0.377849064	TRUE
PP947634.1	B1	0.478608316	0.48052768	0.03857804	0.102207591	B1	1.870684978	2.086187697	0.080274744	0.299569493	B1	0.377423984	0.385806466	0.034188294	0.082141978	TRUE
PP947635.1	A2	0.110328684	0.054096084	0.477971052	0.468023228	A2	0.387120816	0.150302913	2.043001037	2.01638527	A2	0.100643643	0.048934785	0.392737803	0.377836184	TRUE
PP947636.1	A2	0.110712838	0.054480239	0.478355207	0.468407382	A2	0.381653806	0.144835903	2.037534028	2.010918261	A2	0.100645647	0.04893679	0.392739807	0.377838189	TRUE
PP947637.1	B2	0.471917404	0.473836768	0.105464503	0.030557802	B2	1.855004111	2.07050683	0.310504393	0.114006463	B2	0.366282815	0.374665297	0.085902428	0.023271812	TRUE
PP947638.1	A2	0.109609679	0.053377079	0.477252047	0.467304223	A2	0.382903779	0.146085876	2.038784001	2.012168234	A2	0.10003683	0.048327973	0.39213099	0.377229372	TRUE
PP947639.1	B2	0.473837419	0.475756783	0.107384518	0.032477817	B2	1.85815556	2.073658279	0.313655842	0.117157912	B2	0.370077181	0.378459663	0.089696793	0.027066178	TRUE
PP947640.1	B2	0.469303489	0.471222853	0.102850588	0.027943887	B2	1.816025402	2.031528121	0.271525684	0.075027754	B2	0.365789799	0.374172281	0.085409412	0.022778796	TRUE
PP947641.1	A2	0.110684107	0.054451507	0.478326475	0.468378651	A2	0.383049958	0.146232054	2.038930179	2.012314412	A2	0.104405723	0.052696866	0.396499883	0.381598265	TRUE
PP947642.1	A2	0.110990619	0.05475802	0.478632988	0.468685163	A2	0.385154545	0.148336642	2.041034767	2.014419	A2	0.101898606	0.050189748	0.393992766	0.379091148	TRUE
PP947643.1	A2	0.11091216	0.05467956	0.478554528	0.468606704	A2	0.38516292	0.148345017	2.041043142	2.014427375	A2	0.100638227	0.048929369	0.392732387	0.377830769	TRUE
PP947644.1	A2	0.110340767	0.054108167	0.477983136	0.468035311	A2	0.381653806	0.144835903	2.037534028	2.010918261	A2	0.100645647	0.04893679	0.392739807	0.377838189	TRUE
PP947645.1	A2	0.110488882	0.054256283	0.478131251	0.468183426	A2	0.381653806	0.144835903	2.037534028	2.010918261	A2	0.101880339	0.050171482	0.393974499	0.379072881	TRUE
PP947646.1	A2	0.110076486	0.053843886	0.477718854	0.467771029	A2	0.382846256	0.146028352	2.038726477	2.01211071	A2	0.101280815	0.049571957	0.393374975	0.378473356	TRUE
PP947647.1	B1	0.478763852	0.480683217	0.038733577	0.102363127	B1	1.864442224	2.079944943	0.07403199	0.293326739	B1	0.37742259	0.385805072	0.0341869	0.082140584	TRUE
PP947648.1	A2	0.110615767	0.054383167	0.478258135	0.468310311	A2	0.389184634	0.152366731	2.045064856	2.018449089	A2	0.09939521	0.047686352	0.39148937	0.376587751	TRUE
PP947649.1	A2	0.109391654	0.053159054	0.477034022	0.467086197	A2	0.392692884	0.155874981	2.048573105	2.021957338	A2	0.100663328	0.048954471	0.392757488	0.37785587	TRUE
PP947650.1	A2	0.110099282	0.053866682	0.47774165	0.467793826	A2	0.387120816	0.150302913	2.043001037	2.01638527	A2	0.099401122	0.047692264	0.391495282	0.376593663	TRUE
PP947651.1	A2	0.110315017	0.054082418	0.477957386	0.468009561	A2	0.383261237	0.146443334	2.039141458	2.012525691	A2	0.10126227	0.049553413	0.39335643	0.378454812	TRUE
PP947652.1	B1	0.477481781	0.479401145	0.037451505	0.101081056	B1	1.874469698	2.089972417	0.084059464	0.303354213	B1	0.376800303	0.385182785	0.033564613	0.081518297	TRUE
PP947653.1	B1	0.479492553	0.481411918	0.039462278	0.103091828	B1	1.859072151	2.07457487	0.068661917	0.287956666	B1	0.375531567	0.383914049	0.032295877	0.080249561	TRUE
PP947654.1	A2	0.109609679	0.053377079	0.477252047	0.467304223	A2	0.382903779	0.146085876	2.038784001	2.012168234	A2	0.10003683	0.048327973	0.39213099	0.377229372	TRUE
PP947655.1	A2	0.110702662	0.054470062	0.47834503	0.468397205	A2	0.376181129	0.139363226	2.032061351	2.005445584	A2	0.102583152	0.050874294	0.394677312	0.379775693	TRUE

Accession	Sub-type	A1 distance	A2 distance	B1 distance	B2 distance	Sub-type	A1 distance	A2 distance	B1 distance	B2 distance	Sub-type	A1 distance	A2 distance	B1 distance	B2 distance	Concordant
PP947656.1	B1	0.478385345	0.480304709	0.038355069	0.101984619	B1	1.861013053	2.076515772	0.070602819	0.289897567	B1	0.375540578	0.383923059	0.032304887	0.080258572	TRUE
PP947657.1	A2	0.110174799	0.053942199	0.477817168	0.467869343	A2	0.389177983	0.15236008	2.045058205	2.018442437	A2	0.100018343	0.048309486	0.392112503	0.377210885	TRUE
PP947658.1	B2	0.470687625	0.472606989	0.104234724	0.029328023	B2	1.851868112	2.067370832	0.307368394	0.110870464	B2	0.365653538	0.374036019	0.08527315	0.022642535	TRUE
PP947659.1	B2	0.47117006	0.473089425	0.104717159	0.029810459	B2	1.851857554	2.067360273	0.307357836	0.110859906	B2	0.365653538	0.374036019	0.08527315	0.022642535	TRUE
PP947660.1	A2	0.110236547	0.054003947	0.477878915	0.467931091	A2	0.385822309	0.149004406	2.04170253	2.015086763	A2	0.099401122	0.047692264	0.391495282	0.376593663	TRUE
PP947661.1	A2	0.111030608	0.054798008	0.478672976	0.468725152	A2	0.380976347	0.144158444	2.036856569	2.010240802	A2	0.105640522	0.053931665	0.397734682	0.382833064	TRUE
PP947662.1	B1	0.478657865	0.48057723	0.038627589	0.10225714	B1	1.873585153	2.089087872	0.083174919	0.302469668	B1	0.376793795	0.385176277	0.033558105	0.081511789	TRUE
PP947663.1	B2	0.47117113	0.473090494	0.104718229	0.029811528	B2	1.848759685	2.064262404	0.304259966	0.107762036	B2	0.365653538	0.374036019	0.08527315	0.022642535	TRUE
PP947664.1	B1	0.481514476	0.483433841	0.0414842	0.105113751	B1	1.8692863	2.084789019	0.078876066	0.298170815	B1	0.379286676	0.387669157	0.036050986	0.08400467	TRUE
PP947665.1	A2	0.110231618	0.053999018	0.477873986	0.467926161	A2	0.39157556	0.154757657	2.047455782	2.020840015	A2	0.100021156	0.048312298	0.392115316	0.377213697	TRUE
PP947666.1	A2	0.110473897	0.054241298	0.478116266	0.468168441	A2	0.380978912	0.144161009	2.036859133	2.010243366	A2	0.100641366	0.048932509	0.392735526	0.377833908	TRUE
PP947667.1	B1	0.479232123	0.481151487	0.039201847	0.102831397	B1	1.874468524	2.089971243	0.08405829	0.303353039	B1	0.38128977	0.389672251	0.038054079	0.086007764	TRUE
PP947668.1	B2	0.47166491	0.473584275	0.105212009	0.030305309	B2	1.85654268	2.072045399	0.312042962	0.115545032	B2	0.366282815	0.374665297	0.085902428	0.023271812	TRUE
PP947669.1	A2	0.109524011	0.053291411	0.477166379	0.467218555	A2	0.383069222	0.146251319	2.038949444	2.012333677	A2	0.09939204	0.047683182	0.3914862	0.376584581	TRUE
PP947670.1	B2	0.470158264	0.472077628	0.103705363	0.028798662	B2	1.833223752	2.048726472	0.288724034	0.092226104	B2	0.364532831	0.372915312	0.084152443	0.021521827	TRUE
PP947671.1	A2	0.108510109	0.052277509	0.476152477	0.466204653	A2	0.40028598	0.163468077	2.056166202	2.029550435	A2	0.096884815	0.045175957	0.388978975	0.374077356	TRUE
PP947672.1	B2	0.472607554	0.474526918	0.106154653	0.031247952	B2	1.853315257	2.068817976	0.308815539	0.112317609	B2	0.369427983	0.377810465	0.089047596	0.02641698	TRUE
PP947673.1	A2	0.110327613	0.054095014	0.477969982	0.468022157	A2	0.393316642	0.156498738	2.049196863	2.022581096	A2	0.10002839	0.048319533	0.39212255	0.377220932	TRUE
PP947674.1	A2	0.110102773	0.053870174	0.477745142	0.467797317	A2	0.381653806	0.144835903	2.037534028	2.010918261	A2	0.100645647	0.04893679	0.392739807	0.377838189	TRUE
PP947675.1	A2	0.109457422	0.053224823	0.477099791	0.467151966	A2	0.382903779	0.146085876	2.038784001	2.012168234	A2	0.10003683	0.048327973	0.39213099	0.377229372	TRUE
PP947676.1	A2	0.111278121	0.055045521	0.478920489	0.468972665	A2	0.380987899	0.144169996	2.036868121	2.010252354	A2	0.101893076	0.050184218	0.393987236	0.379085617	TRUE
PP947677.1	A2	0.110055184	0.053822584	0.477697552	0.467749728	A2	0.382903779	0.146085876	2.038784001	2.012168234	A2	0.10003683	0.048327973	0.39213099	0.377229372	TRUE
PP947678.1	B2	0.470314115	0.472233479	0.103861214	0.028954513	B2	1.836228435	2.051731154	0.291728717	0.095230787	B2	0.364532831	0.372915312	0.084152443	0.021521827	TRUE
PP947679.1	B1	0.479802801	0.481722166	0.039772526	0.103402076	B1	1.859349201	2.07485192	0.068938967	0.288233715	B1	0.376779559	0.38516204	0.033543868	0.081497553	TRUE
PP947680.1	B2	0.471017287	0.472936652	0.104564387	0.029657686	B2	1.839856276	2.055358995	0.295356558	0.098858628	B2	0.363931923	0.372314405	0.083551535	0.02092092	TRUE
PP947681.1	B2	0.470158269	0.472077634	0.103705369	0.028798668	B2	1.834718863	2.050221583	0.290219145	0.093721215	B2	0.364532831	0.372915312	0.084152443	0.021521827	TRUE
PP947682.1	B2	0.470232774	0.472152139	0.103779873	0.028873173	B2	1.833223752	2.048726472	0.288724034	0.092226104	B2	0.365151659	0.373534141	0.084771272	0.022140656	TRUE
PP947683.1	B2	0.470694199	0.472613564	0.104241298	0.029334598	B2	1.848759685	2.064262404	0.304259966	0.107762036	B2	0.365653538	0.374036019	0.08527315	0.022642535	TRUE
PP947684.1	B1	0.479304011	0.481223375	0.039273735	0.102903285	B1	1.866081777	2.081584496	0.075671544	0.294966292	B1	0.378041432	0.386423914	0.034805742	0.082759426	TRUE
PP947685.1	A2	0.109769808	0.053537208	0.477412176	0.467464352	A2	0.393605156	0.156787252	2.049485377	2.02286961	A2	0.100014508	0.04830565	0.392108668	0.377207049	TRUE
PP947686.1	B2	0.467931708	0.469851072	0.101478807	0.030447587	B2	1.842007431	2.05751015	0.297507713	0.101009783	B2	0.370136393	0.378518875	0.089756006	0.02712539	TRUE
PP947687.1	A2	0.11107594	0.05484334	0.478718308	0.468770484	A2	0.38731611	0.150498207	2.043196332	2.016580565	A2	0.098777184	0.047068326	0.390871344	0.375969725	TRUE
PP947688.1	B1	0.479637816	0.48155718	0.03960754	0.103237091	B1	1.874677686	2.090180405	0.084267452	0.303562201	B1	0.377427101	0.385809583	0.034191411	0.082145095	TRUE
PP947689.1	B2	0.47166491	0.473584275	0.105212009	0.030305309	B2	1.85654268	2.072045399	0.312042962	0.115545032	B2	0.366282815	0.374665297	0.085902428	0.023271812	TRUE
PP947690.1	B2	0.47199578	0.473915144	0.105542879	0.030636178	B2	1.831545282	2.047048001	0.287045564	0.090547633	B2	0.365155372	0.373537854	0.084774985	0.022144369	TRUE
PP947691.1	B2	0.471111807	0.473031172	0.104658906	0.029752206	B2	1.834475613	2.049978332	0.289975895	0.093477965	B2	0.367058184	0.375440666	0.086677796	0.024047181	TRUE
PP947692.1	A2	0.110542975	0.054310376	0.478185344	0.468237519	A2	0.388544999	0.151727096	2.044425221	2.017809454	A2	0.100642519	0.048933661	0.392736679	0.37783506	TRUE
PP947693.1	A2	0.110013175	0.053780576	0.477655544	0.467707719	A2	0.385821613	0.149003709	2.041701834	2.015086067	A2	0.099401122	0.047692264	0.391495282	0.376593663	TRUE
PP947694.1	B1	0.47942569	0.481345055	0.039395414	0.103024965	B1	1.882585591	2.09808831	0.092175357	0.311470105	B1	0.377422123	0.385804604	0.034186433	0.082140117	TRUE
PP947695.1	B1	0.479508953	0.481428317	0.039478677	0.103108228	B1	1.871669654	2.087172373	0.08125942	0.300554169	B1	0.378051564	0.386434046	0.034815874	0.082769558	TRUE
PP947696.1	A2	0.110794008	0.054561409	0.478436377	0.468488552	A2	0.391761596	0.154943693	2.047641818	2.021026051	A2	0.097524445	0.045815587	0.389618605	0.374716987	TRUE
PP947697.1	B2	0.469660287	0.471579651	0.103207386	0.028300685	B2	1.806913182	2.022415901	0.262413463	0.065915533	B2	0.365789799	0.374172281	0.085409412	0.022778796	TRUE
PP947698.1	A2	0.109672201	0.053439601	0.47731457	0.467366745	A2	0.383069222	0.146251319	2.038949444	2.012333677	A2	0.09939204	0.047683182	0.3914862	0.376584581	TRUE
PP947699.1	A2	0.110956342	0.054723743	0.478598711	0.468650886	A2	0.380976347	0.144158444	2.036856569	2.010240802	A2	0.105641274	0.053932416	0.397735434	0.382833815	TRUE
PP947700.1	A2	0.109319455	0.053086855	0.476961824	0.467013999	A2	0.383060144	0.146242241	2.038940366	2.012324598	A2	0.100016081	0.048307224	0.392110241	0.377208623	TRUE
PP947701.1	B1	0.47915919	0.481078554	0.039128914	0.102758465	B1	1.865948628	2.081451347	0.075538394	0.294833142	B1	0.378041914	0.386424396	0.034806224	0.082759908	TRUE
PP947702.1	B2	0.471091926	0.47301129	0.104639025	0.029732324	B2	1.848759685	2.064262404	0.304259966	0.107762036	B2	0.366907664	0.375290146	0.086527276	0.023896661	TRUE
PP947703.1	B2	0.471331192	0.473250557	0.104878292	0.029971591	B2	1.850304958	2.065807677	0.305805239	0.109307309	B2	0.366906669	0.37528915	0.086526281	0.023895665	TRUE
PQ634884.1	A2	0.110460786	0.054228186	0.478103154	0.46815533	A2	0.383724572	0.146906669	2.039604794	2.012989027	A2	0.101261682	0.049552825	0.393355842	0.378454224	TRUE
PQ634888.1	A2	0.109829801	0.053597202	0.47747217	0.467524345	A2	0.380987667	0.144169764	2.036867889	2.010252122	A2	0.101281568	0.04957271	0.393375728	0.378474109	TRUE
PQ634896.1	A2	0.113139636	0.056907036	0.480782004	0.470834179	A2	0.394933785	0.158115882	2.050814007	2.02419824	A2	0.103136429	0.051427571	0.395230589	0.38032897	TRUE
PQ634898.1	A2	0.1099408	0.053708201	0.477583169	0.467635344	A2	0.384387036	0.147569133	2.040267258	2.013651491	A2	0.100645647	0.04893679	0.392739807	0.377838189	TRUE

Accession	Sub-type	A1 distance	A2 distance	B1 distance	B2 distance	Sub-type	A1 distance	A2 distance	B1 distance	B2 distance	Sub-type	A1 distance	A2 distance	B1 distance	B2 distance	Concordant
PQ634901.1	A2	0.11098724	0.05475464	0.478629608	0.468681784	A2	0.39704131	0.160223407	2.052921532	2.026305765	A2	0.101259067	0.049550209	0.393353227	0.378451609	TRUE
PQ634925.1	A2	0.110026611	0.053794011	0.477668979	0.467721154	A2	0.387120816	0.150302913	2.043001037	2.01638527	A2	0.100016187	0.048307329	0.392110347	0.377208728	TRUE
PQ634931.1	A2	0.110325646	0.054093046	0.477968015	0.46802019	A2	0.389184041	0.152366138	2.045064263	2.018448496	A2	0.10002839	0.048319533	0.39212255	0.377220932	TRUE
PQ634937.1	A2	0.113009777	0.056777177	0.480652145	0.47070432	A2	0.394399569	0.157581666	2.050279791	2.023664023	A2	0.105015855	0.053306997	0.397110015	0.382208396	TRUE
PQ634938.1	A2	0.110402069	0.054169469	0.478044437	0.468096613	A2	0.387120816	0.150302913	2.043001037	2.01638527	A2	0.099401122	0.047692264	0.391495282	0.376593663	TRUE
PQ634941.1	A2	0.110615269	0.054382669	0.478257638	0.468309813	A2	0.395731976	0.158914073	2.051612198	2.024996431	A2	0.097528317	0.045819459	0.389622477	0.374720859	TRUE
PQ634949.1	A2	0.1112251	0.0549925	0.478867469	0.468919644	A2	0.385111173	0.14829327	2.040991395	2.014375628	A2	0.103164919	0.051456062	0.395259079	0.380357461	TRUE
PQ634950.1	A2	0.110910006	0.054677406	0.478552374	0.46860455	A2	0.392893182	0.156075278	2.048773403	2.022157636	A2	0.10069037	0.048981513	0.39278453	0.377882912	TRUE
PQ634956.1	A2	0.113914825	0.057682226	0.481557194	0.471609369	A2	0.396846418	0.160028515	2.05272664	2.026110873	A2	0.104380414	0.052671557	0.396474574	0.381572956	TRUE
PQ634962.1	A2	0.11032393	0.05409133	0.477966298	0.468018474	A2	0.389177844	0.152359941	2.045058066	2.018442298	A2	0.100636859	0.048928002	0.392731019	0.377829401	TRUE
PV081662.1	A2	0.120587918	0.064355318	0.488230286	0.478282462	A2	0.460598986	0.223781082	2.116479207	2.08986344	A2	0.108800225	0.057091367	0.400894385	0.385992766	TRUE
PV081663.1	B2	0.476464283	0.478383647	0.110011382	0.035104681	B2	1.854730618	2.070233337	0.3102309	0.11373297	B2	0.368933705	0.377316186	0.088553317	0.025922702	TRUE
PV081664.1	B2	0.474549645	0.476469009	0.108096744	0.033190043	B2	1.842291177	2.057793896	0.297791459	0.101293529	B2	0.367055123	0.375437605	0.086674736	0.02404412	TRUE
PV081665.1	A2	0.112995291	0.056762692	0.48063766	0.470689835	A2	0.197701539	0.191493007	1.853581761	1.826965993	A2	0.101916257	0.0502074	0.394010417	0.379108799	TRUE
PV081666.1	A2	0.118161381	0.061928781	0.48580375	0.475855925	A2	0.44815933	0.211341427	2.104039552	2.077423785	A2	0.105862104	0.054153247	0.397956264	0.383054646	TRUE
PV081667.1	B2	0.476746033	0.478665397	0.110293132	0.035386431	B2	1.879730438	2.095233157	0.33523072	0.13873279	B2	0.373272086	0.381654567	0.092891698	0.030261082	TRUE
PV353969.1	A2	0.120822581	0.064589981	0.488464949	0.478517125	A2	0.416119611	0.179301708	2.071999833	2.045384066	A2	0.105622201	0.053913343	0.397716361	0.382814742	TRUE
PV353970.1	A2	0.115562246	0.059329646	0.483204614	0.47325679	A2	0.405510514	0.168692611	2.061390736	2.034774969	A2	0.103760203	0.052051346	0.395854363	0.380952745	TRUE
PV353971.1	A2	0.114031986	0.057799386	0.481674354	0.47172653	A2	0.399263409	0.162445506	2.055143631	2.028527864	A2	0.103136429	0.051427571	0.395230589	0.38032897	TRUE
PV353972.1	A2	0.114032087	0.057799487	0.481674455	0.471726631	A2	0.401315466	0.164497563	2.057195688	2.030579921	A2	0.103757231	0.052048373	0.395851391	0.380949772	TRUE
PV353973.1	A2	0.114780901	0.058548301	0.482423269	0.472475444	A2	0.405443619	0.168625716	2.061323841	2.034708074	A2	0.105000207	0.05329135	0.397094367	0.382192749	TRUE
PV353974.1	A2	0.115234114	0.059001514	0.482876482	0.472928658	A2	0.411882233	0.17506433	2.067762455	2.041146688	A2	0.105003582	0.053294724	0.397097742	0.382196123	TRUE
PV353975.1	A2	0.115234114	0.059001514	0.482876482	0.472928658	A2	0.411882233	0.17506433	2.067762455	2.041146688	A2	0.105003582	0.053294724	0.397097742	0.382196123	TRUE
PV353976.1	A2	0.115304891	0.059072291	0.48294726	0.472999435	A2	0.403383397	0.166565494	2.059263619	2.032647852	A2	0.105001833	0.053292975	0.397095993	0.382194375	TRUE
PV353977.1	A2	0.114405719	0.058173119	0.482048087	0.472100263	A2	0.403391267	0.166573363	2.059271488	2.032655721	A2	0.104999501	0.053290644	0.397093661	0.382192043	TRUE
PV353978.1	B1	0.480049728	0.481969092	0.040019452	0.103649002	B1	1.878005022	2.093507742	0.087594789	0.306889537	B1	0.374323285	0.382705766	0.031087594	0.079041279	TRUE
PV660306.1	A2	0.115854923	0.059622324	0.483497292	0.473549467	A2	0.405608117	0.168790214	2.061488339	2.034872572	A2	0.105648884	0.053940026	0.397743044	0.382841426	TRUE
PV660307.1	A2	0.115241022	0.059008422	0.48288339	0.472935566	A2	0.405607712	0.168789809	2.061487934	2.034872167	A2	0.105632219	0.053923362	0.397726379	0.382824761	TRUE
PV660308.1	B2	0.474326524	0.476245888	0.107873623	0.032966922	B2	1.858021061	2.07352378	0.313521342	0.117023412	B2	0.371309296	0.379691777	0.090928908	0.028298292	TRUE
PV660309.1	A2	0.115241022	0.059008422	0.48288339	0.472935566	A2	0.405607712	0.168789809	2.061487934	2.034872167	A2	0.105632219	0.053923362	0.397726379	0.382824761	TRUE
PV660310.1	B1	0.48277456	0.484693924	0.042744284	0.106373834	B1	1.887896891	2.10339961	0.097486658	0.316781406	B1	0.379913601	0.388296082	0.036677911	0.084631595	TRUE
PV660311.1	B2	0.47460399	0.476523354	0.108151089	0.033244388	B2	1.859566391	2.07506911	0.315066673	0.118568743	B2	0.370675082	0.379057563	0.090294694	0.027664078	TRUE
PX120316.1	B1	0.482838731	0.484758096	0.042808456	0.106438006	B1	1.866492957	2.081995676	0.076082723	0.295377472	B1	0.379289378	0.38767186	0.036053688	0.084007372	TRUE
PX120317.1	A2	0.115769135	0.059536535	0.483411503	0.473463679	A2	0.412136936	0.175319033	2.068017158	2.041401391	A2	0.10274243	0.051033572	0.39483659	0.379934971	TRUE
PX120318.1	A2	0.114960653	0.058728054	0.482603022	0.472655197	A2	0.409444785	0.172626881	2.065325006	2.038709239	A2	0.105847777	0.054138919	0.397941937	0.383040318	TRUE
PX120319.1	A2	0.115120856	0.058888257	0.482763225	0.4728154	A2	0.416062711	0.179244808	2.071942933	2.045327166	A2	0.103973235	0.052264377	0.396067395	0.381165777	TRUE
PX120320.1	A2	0.114051001	0.057818401	0.481693369	0.471745545	A2	0.406255149	0.169437246	2.062135371	2.035519604	A2	0.102734734	0.051025876	0.394828894	0.379927276	TRUE
PX120321.1	A2	0.113581586	0.057348986	0.481223954	0.47127613	A2	0.265913207	0.163627706	1.921793429	1.895177662	A2	0.10001249	0.048303632	0.39210665	0.377205032	TRUE
PX120322.1	A2	0.113844289	0.057611689	0.481486657	0.471538833	A2	0.268913136	0.166627634	1.924793357	1.89817759	A2	0.10001249	0.048303632	0.39210665	0.377205032	TRUE
PX120323.1	A2	0.114178611	0.057946012	0.48182098	0.471873155	A2	0.274450718	0.172165217	1.93033094	1.903715173	A2	0.102535241	0.050826383	0.394629401	0.379727782	TRUE

Figure S1. Pairwise agreement matrices comparing hMPV F gene-based subtype assignments with G gene- and whole-genome-based assignments.

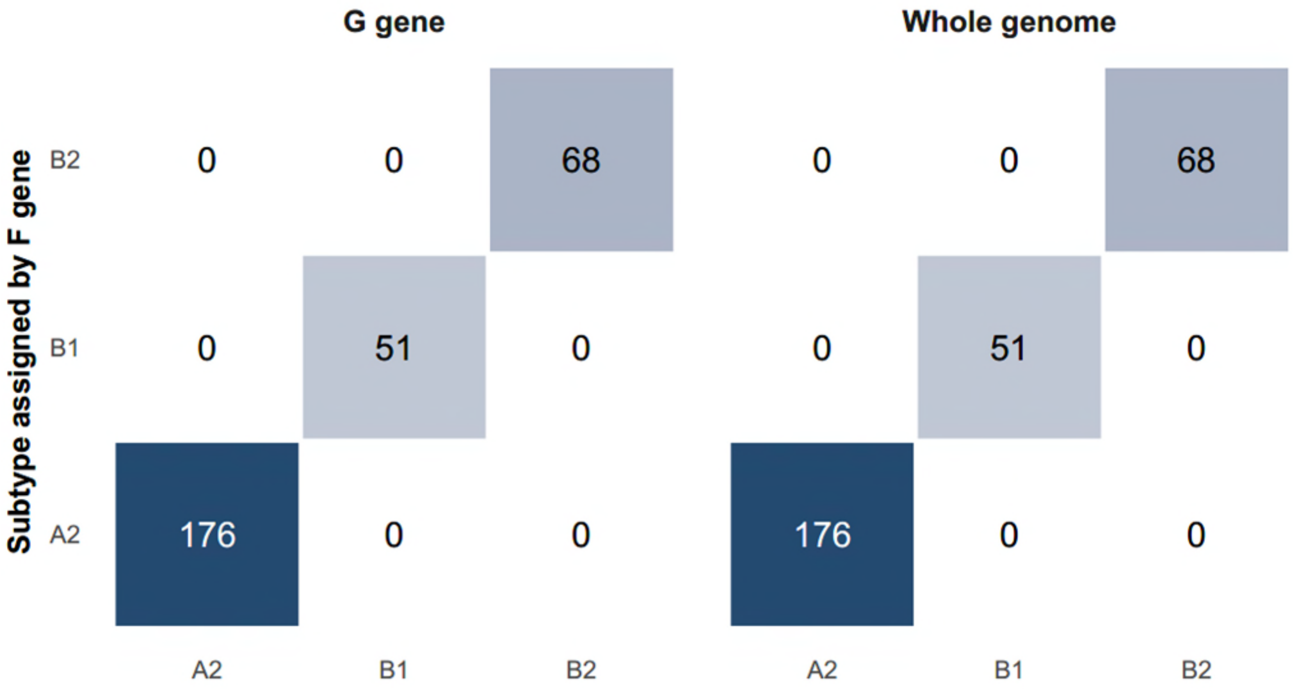

Rows represent F gene subtype assignments and columns represent subtype assignments from the comparison method. Values indicate the number of isolates in each cell. All 295 isolates were located on the diagonal in both comparisons, with no discordant assignments.
F, fusion protein; G, glycoprotein; hMPV, human metapneumovirus.

Figure S2. (**A**) Total number of hMPV sequences in the public surveillance dataset between 1982 and 2025 (N = 1646) by country of origin, and (**B**) hMPV subtype proportion by country of origin.

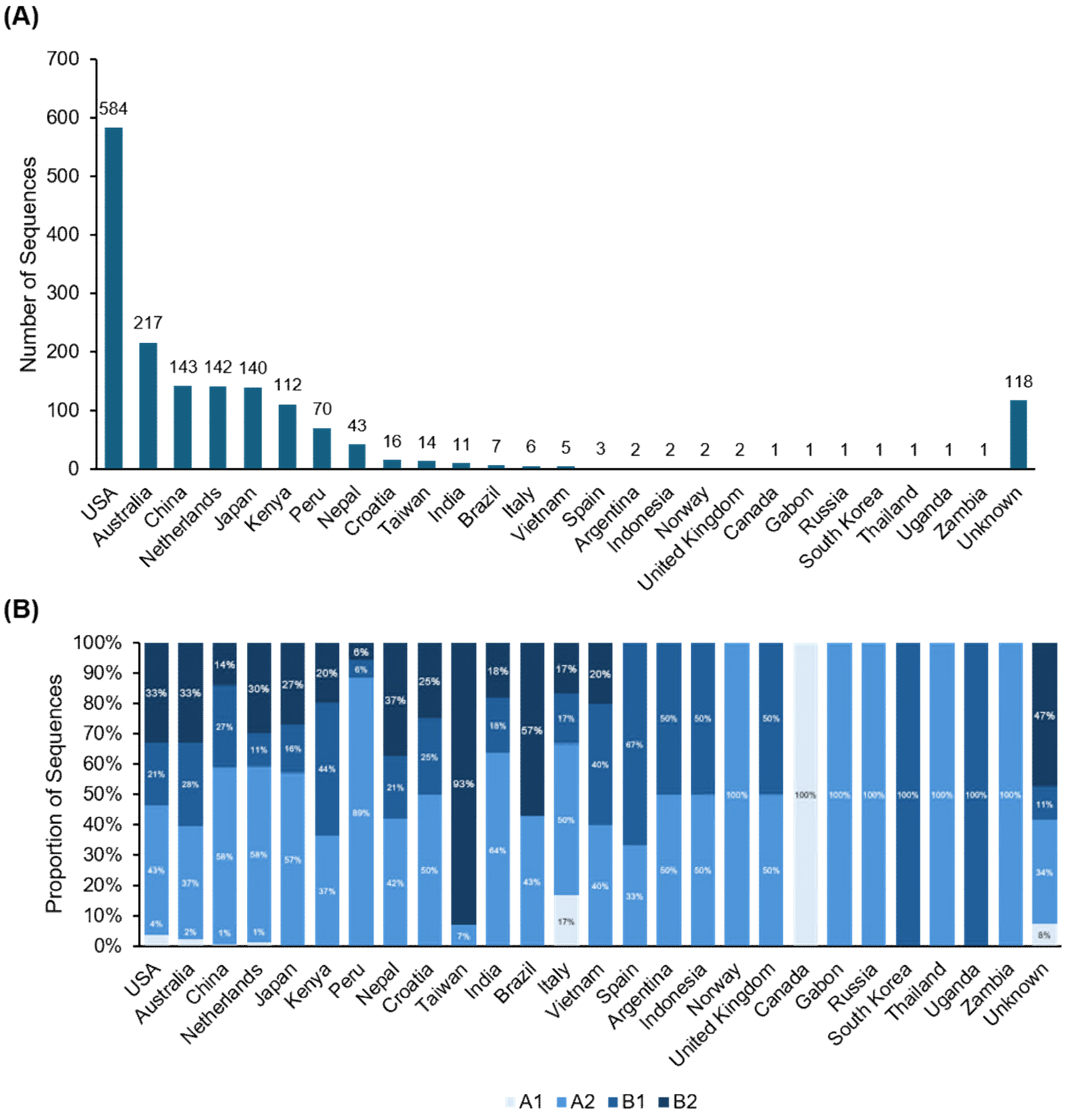

hMPV, human metapneumovirus.

Figure S3. Phylogenetic trees of (**A**) all hMPV F gene sequences in the combined contemporary data set (*N* = 724), and (**B**) based on source of sequences (contemporary public surveillance dataset versus contemporary nasal swab specimen dataset).

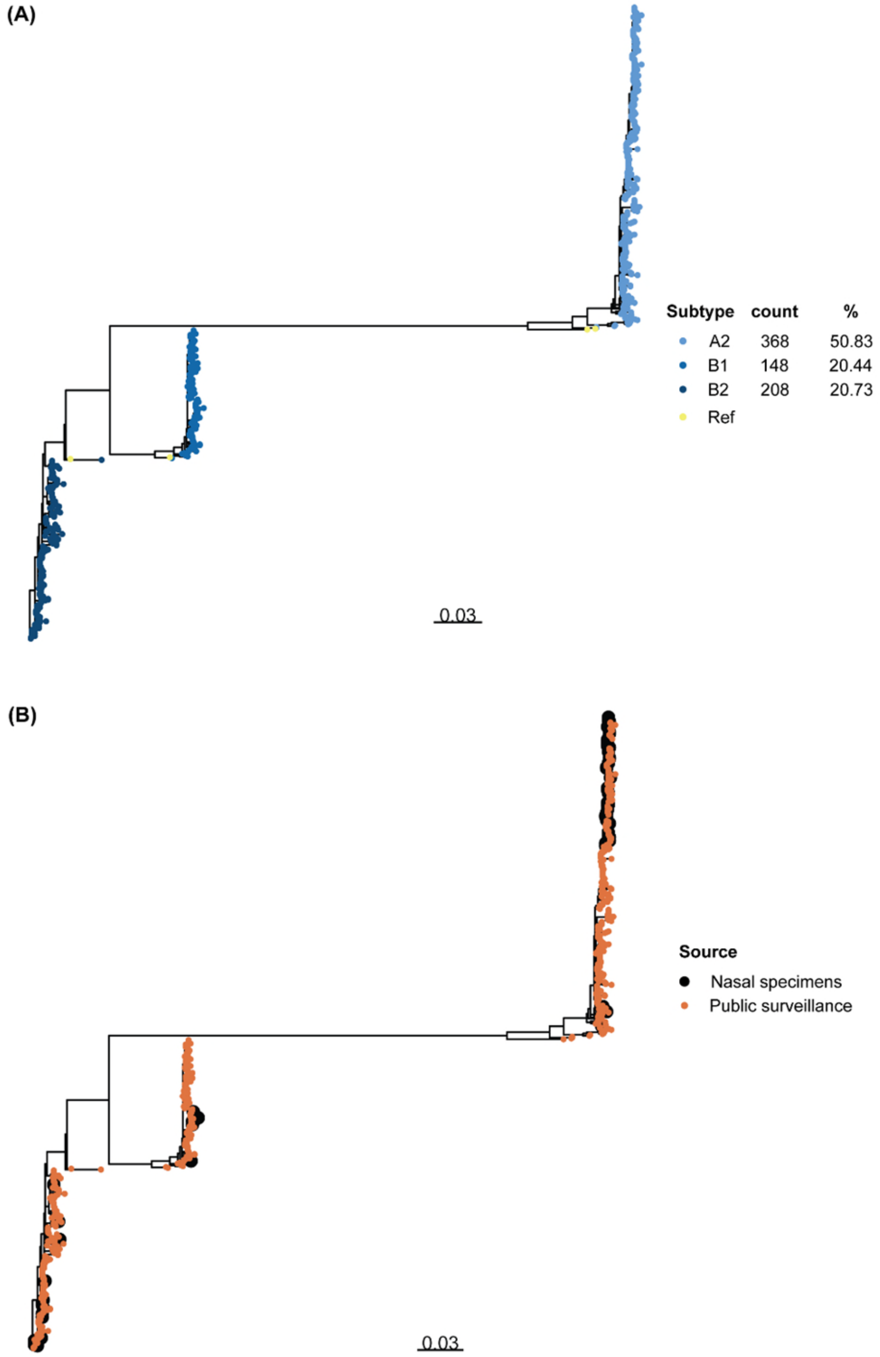

A1 ref: 00-1 [accession: NC_039199.1], A2 ref: NL/00/17 [accession: FJ168779.1], B1 ref: NL/1/99 [accession: AY525843.1], B2 ref: NL/94/01 [accession: FJ168778.1].

F, fusion protein; hMPV, human metapneumovirus.

Figure S4. Pairwise phylogenetic distances among contemporary hMPV F sequences.

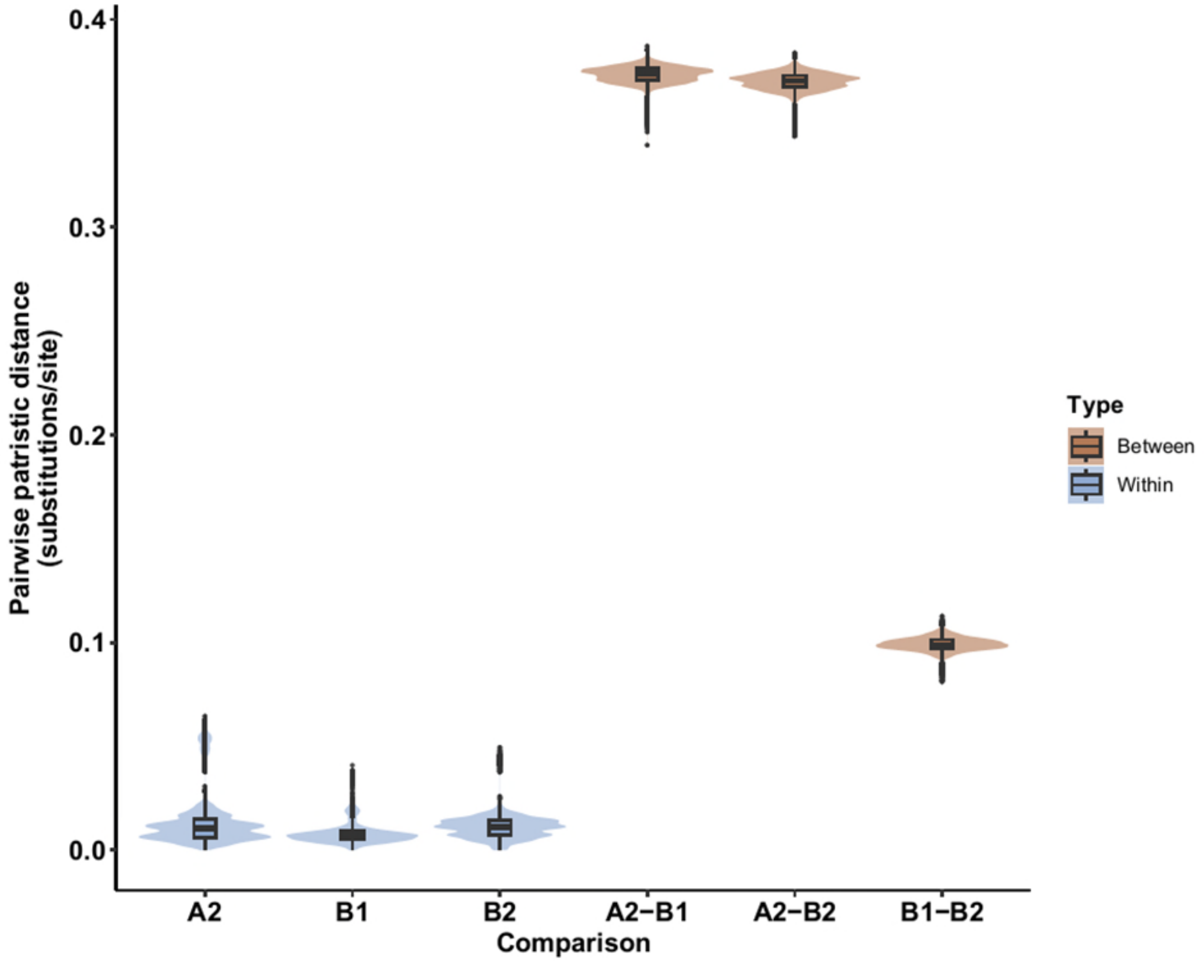

F, fusion protein; hMPV, human metapneumovirus.

Figure S5. Per-codon selection across the hMPV F gene estimated by FEL (*N* = 722; IQ-TREE 2 phylogeny).

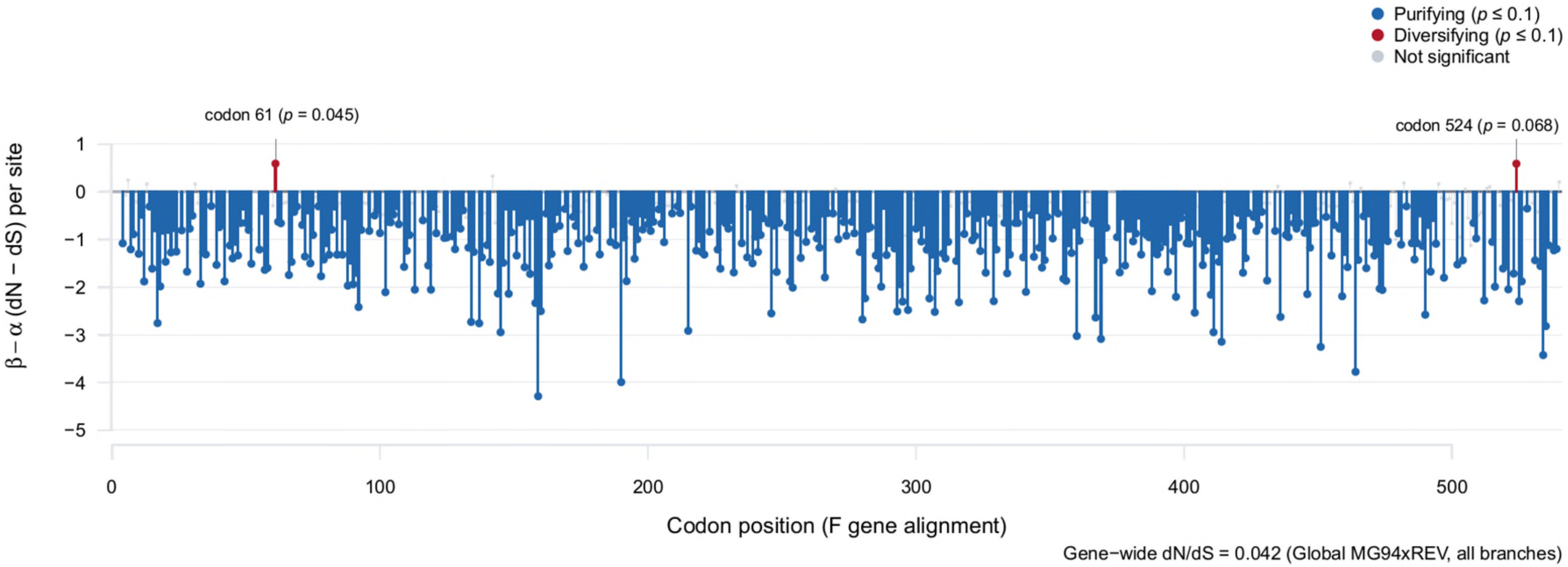

Below 0 = purifying (blue), above 0 = diversifying (red); grey = not significant.

F, fusion protein; FEL, Fixed Effects Likelihood; hMPV, human metapneumovirus.

Figure S6. hMPV subtype distribution in historical (pre-2017, N = 489[a]) and contemporary (N = 724) datasets.

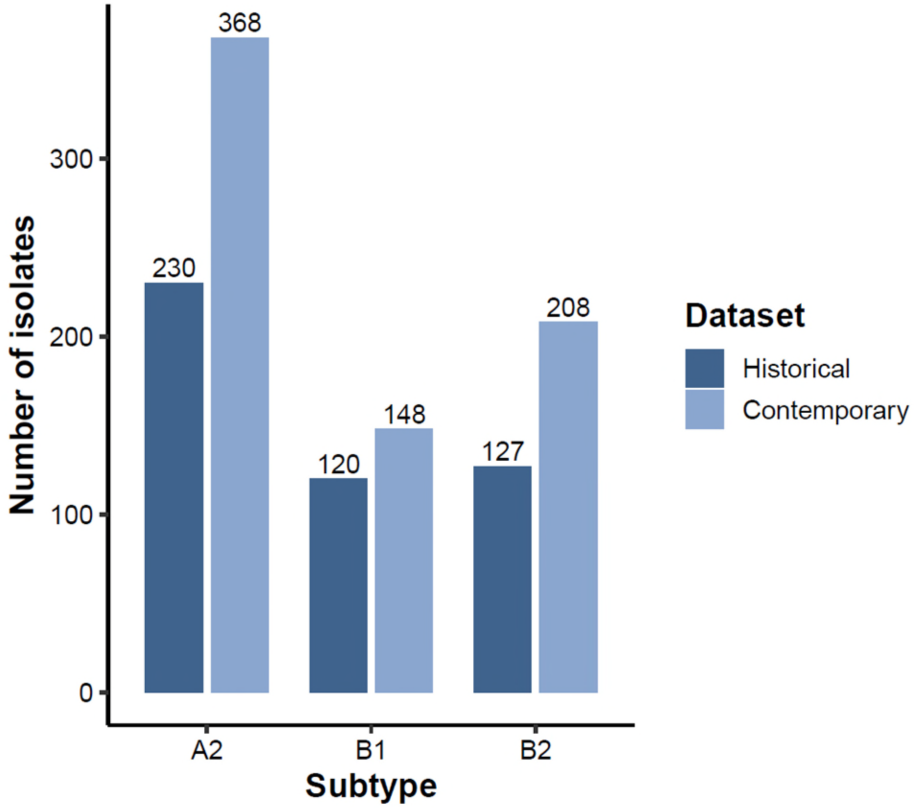

[a]In the historical dataset, 12 sequences were subtype A1.

hMPV, human metapneumovirus.
